# Nucleophilicities and Nucleofugalities of Thio‐ and Selenoethers

**DOI:** 10.1002/chem.202100977

**Published:** 2021-06-30

**Authors:** Biplab Maji, Xin‐Hua Duan, Patrick M. Jüstel, Peter A. Byrne, Armin R. Ofial, Herbert Mayr

**Affiliations:** ^1^ Department Chemie Ludwig-Maximilians-Universität München Butenandtstr. 5–13 81377 München Germany; ^2^ Department of Chemical Sciences Indian Institute of Science Education and Research Kolkata Mohanpur 741246 India; ^3^ Department of Chemistry School of Chemistry Xi'an Key Laboratory of Sustainable Energy Material Chemistry Xi'an Jiaotong University No. 28, Xianning West Road Xi'an 710049 P. R. China; ^4^ School of Chemistry University College Cork College Road Cork Ireland

**Keywords:** kinetics, Lewis bases, linear free energy relationships, thermodynamics, thioethers

## Abstract

Rate constants for the reactions of dialkyl chalcogenides with laser flash photolytically generated benzhydrylium ions have been measured photometrically to integrate them into the comprehensive benzhydrylium‐based nucleophilicity scale. Combining these rate constants with the previously reported equilibrium constants for the same reactions provided the corresponding Marcus intrinsic barriers and made it possible to quantify the leaving group abilities (nucleofugalities) of dialkyl sulfides and dimethyl selenide. Due to the low intrinsic barriers, dialkyl chalcogenides are fairly strong nucleophiles (comparable to pyridine and *N*‐methylimidazole) as well as good nucleofuges; this makes them useful group‐transfer reagents.

## Graphical abstract

**Strong nucleophiles and weak Lewis bases**: Kinetic studies by laser flash photolytic methods reveal that dialkyl sulfides and selenides are strong nucleophiles, comparable to pyridine and *N*‐methylimidazole. Because of their low Lewis basicity (6 to 7 orders of magnitude lower than that of pyridine with respect to C‐centred Lewis acids) they are also good nucleofuges, which makes them useful group‐transfer reagents. Low Marcus intrinsic barriers account for the different ranking of dialkyl chalcogenides in nucleophilicity and Lewis basicity scales.

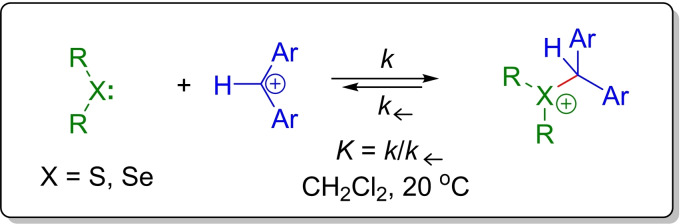

## Introduction

**Scheme 1:**
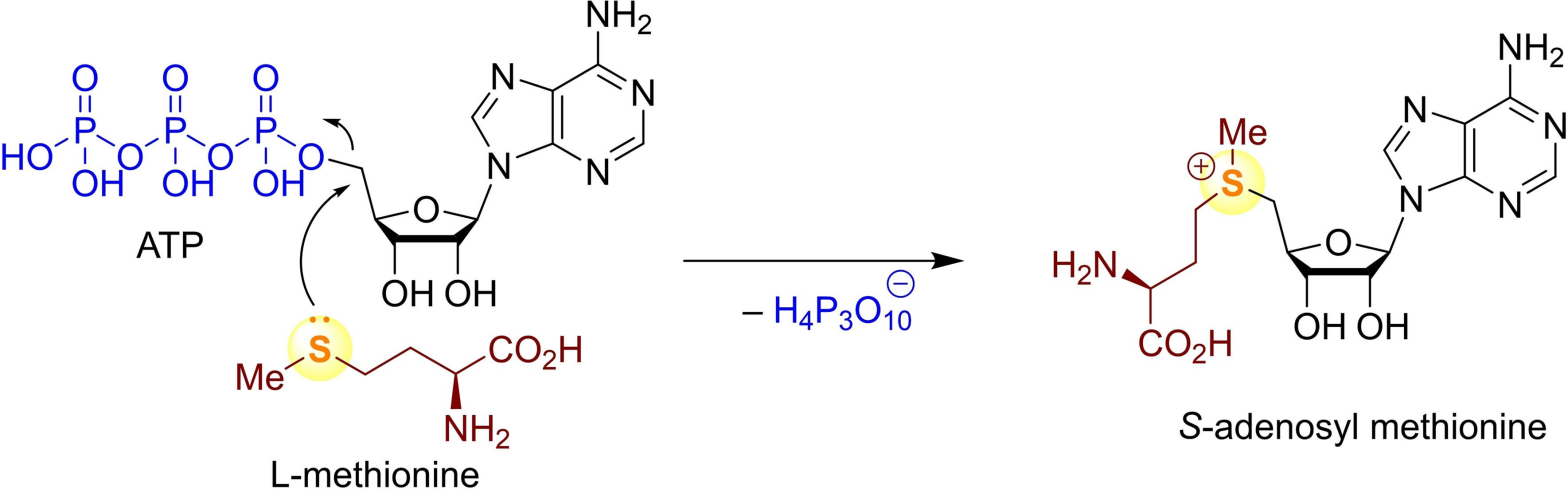
Participation of S‐centred nucleophiles in biological systems.

Dialkyl chalcogenides are known to act as nucleophiles in a variety of reactions.^[1]^ The amino acid l‐methionine, for example, reacts as a sulfur‐centred nucleophile at the 5’‐position of adenosyl triphosphate (ATP) to form *S*‐adenosyl methionine (Scheme 1), which functions as a methylating agent in living organisms.^[2]^ In several hydrolases, the chalcogen‐containing amino acids serine, cysteine, and selenocysteine are essential, and their catalytic activities rely on the nucleophilic properties of the chalcogen atom.^[3]^ Dialkyl chalcogenides have successfully been employed as nucleophilic organocatalysts in various chalcogenide‐ylide mediated reactions, for example, epoxidations, aziridinations, cyclopropanations, and olefinations.^[4]^ Chalcogenides were also used in combination with Lewis acids as organocatalysts in Morita‐Baylis‐Hillman reactions.^[5]^

Arnett has shown that the relative Brønsted basicities of chalcogenides are strongly dependent on the nature of the solvent.^[6]^ While in aqueous solution, dimethyl sulfide (p*K*_aH_=−6.95) is a much weaker base than dimethyl ether (p*K*_aH_=−2.52), the heat of protonation is the same for both compounds in HSO_3_F. In the gas phase, the proton affinity of dimethyl sulfide (197 kcal mol^−1^) is significantly higher than that of dimethyl ether (186 kcal mol^−1^).^[6]^ Similar p*K*_aH_ values for cyclic sulfides have been measured by Scorrano.^[7]^ Because of the well‐known limitations of the correlations between the reactivities of different types of nucleophiles and the corresponding Brønsted basicities,^[8]^ we have previously used benzhydrylium ions as reference electrophiles and reference Lewis acids for the construction of comprehensive nucleophilicity^[9]^ and Lewis basicity scales.^[10]^ Variation of the *p*‐ and *m*‐substituents of the benzhydrylium ions allowed a wide variation of their electrophilicities and Lewis acidities while the steric demand in the vicinity of the reaction centre is kept constant. During earlier work, we have demonstrated that the rates of the reactions of *n*‐, *π*‐, and *σ*‐nucleophiles with carbocations and Michael acceptors can be described by the linear‐free‐energy relationship [Eq. 1].^[9]^

(1) $$\log\;k\left(20\;{}^{\circ }C\right)=s{}_{N}\left(N+E\right)$$

**Scheme 2:**
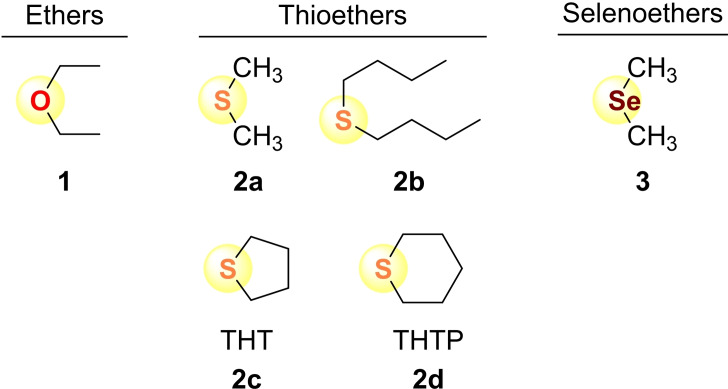
Dialkyl chalcogenides **1**–**3** used in this study.

In Equation (1), nucleophiles are characterized by the solvent‐dependent nucleophilicity parameter *N* and the susceptibility parameter *s*_N_, while electrophiles are characterized by the solvent‐independent electrophilicity parameter *E*.^[9]^ As explained in detail previously,^[9b]^ the unconventional expression of the linear free energy relationship (1) avoids far‐reaching extrapolations by defining nucleophilicity *N* as the negative intercept on the abscissa in log *k* versus *E* plots. Based on Equation (1), the nucleophilicities of numerous *n*‐, *π*‐, and *σ*‐nucleophiles, including amines, pyridines, imidazoles, imidazolines, and phosphanes, have been quantified.^[9d]^ In this work, we set out to determine the nucleophilicities of dialkyl chalcogenides **1**–**3** (Scheme 2) using the benzhydrylium ions **4** depicted in Table 1 as reference electrophiles.

**Table 1:** Electrophilicity (*E*), electrofugality (*E*_f_), and Lewis acidity (*LA*) parameters of the benzhydrylium ions used in this work.

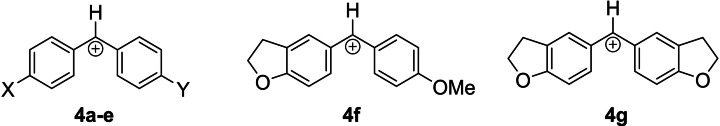						
	X	Y	Abbreviations	*E* ^[a]^	*E*_f_^[b]^	*LA* ^[c]^
**4 a**	OPh	H	(pop)(Ph)CH^+^	2.90	−3.52	4.42
**4 b**	OMe	H	(ani)(Ph)CH^+^	2.11	−2.09	3.10
**4 c**	OMe	Me	(ani)(tol)CH^+^	1.48	−1.32	2.00
**4 d**	OMe	OPh	(ani)(pop)CH^+^	0.61	−0.86	0.90
**4 e**	OMe	OMe	(ani)_2_CH^+^	0	0	0
**4 f**			(fur)(ani)CH^+^	−0.81	0.61	(−1.11)^[d]^
**4 g**			(fur)_2_CH^+^	−1.36	1.07	−1.29

[a] Electrophilicity parameters *E* for benzhydrylium ions from refs. [9] and [11]. [b] Electrofugality parameters *E*_f_ for benzhydrylium ions from ref. [12]. [c] Lewis acidity *LA* of benzhydrylium ions in dichloromethane, from ref. [10b]. [d] Interpolated Lewis acidity *LA*, see ref. [10b].

## Results and Discussion

### Kinetics of the reactions of dialkyl chalcogenides 1–3 with benzhydrylium Ions 4

To characterize the sulfonium ions **4**‐SR_2_ generated by the reactions of the thioethers **2 a**–**d** with benzhydrylium ions, benzhydrylium triflate **4 e**‐OTf (generated by mixing **4 e**‐Cl with trimethylsilyl triflate) was combined with **2 a**–**d** in dichloromethane. As specified by the NMR spectroscopic analysis of the crude products (see the Supporting Information) the corresponding dialkylbenzhydrylsulfonium triflates (**4**‐SR_2_)⋅TfO^−^ were formed exclusively.

**Scheme 3:**
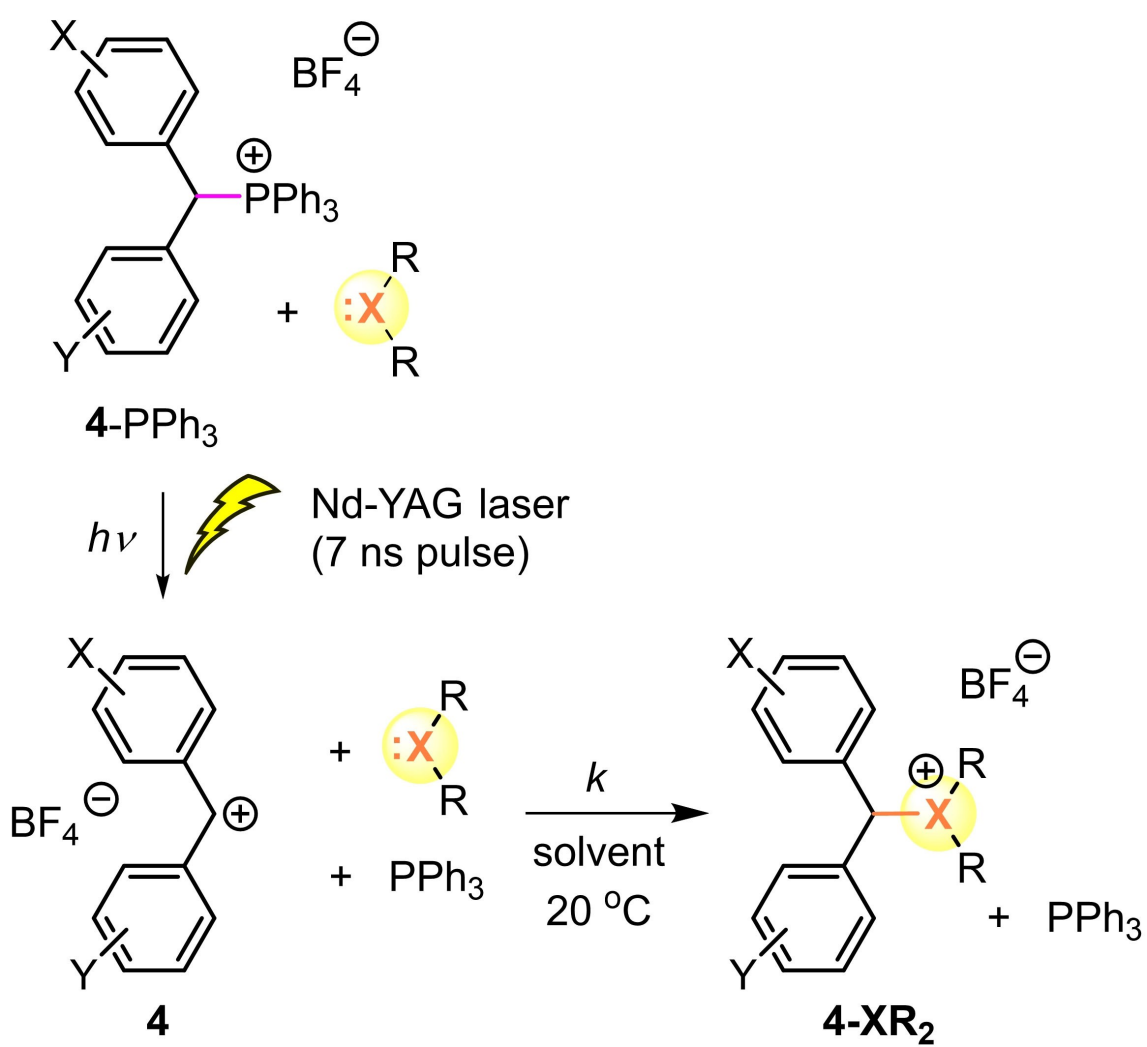
Generation of benzhydrylium ions **4** by laser‐flash irradiation of the precursor phosphonium tetrafluoroborates **4**‐PPh_3_BF_4_ and their combination with dialkyl chalcogenides (at 20 °C in MeCN or CH_2_Cl_2_).

Previous work showed that due to the low Lewis basicities of **1**–**3** their combinations with benzhydrylium ions, which are better stabilized than **4 g** (Lewis acidity *LA*<−2), do not lead to adduct formation in 2 mM dichloromethane solutions.^[10a]^ On the other side, reactions of thio‐ and selenoethers with **4 g** and less stabilized benzhydrylium ions (**4 a**–**f**, *LA*>−1) were so fast that conventional UV/Vis spectroscopy, even when combined with stopped‐flow techniques, was not suitable to follow the decay of the absorbances of the benzhydrylium ions **4** because the reactions are faster than the mixing time in the stopped‐flow instrument. For that reason, laser‐flash photolytic techniques as described previously^[13]^ were employed to study the kinetics of the reactions of diethyl ether (**1**) and the thio‐ and selenoethers **2** and **3** with the benzhydrylium ions **4** (Scheme 3).

The benzhydrylium ions **4** were generated by laser flash irradiation (7 ns pulse, 266 nm, 40–60 mJ/pulse) of the benzhydryltriphenylphosphonium tetrafluoroborates **4**‐PPh_3_BF_4_ in the presence of the nucleophiles **2** or **3** in acetonitrile or dichloromethane at 20 °C. The intermediate benzhydrylium ions **4** were identified by their UV/Vis spectra.^[13]^ The rates of the combination reactions were followed by monitoring the decay of the absorbances of **4** at or close to their absorption maxima.

Quantitative kinetic measurements were not carried out with diethyl ether (**1**) as the addition of two equivalents of **1** to a 0.035 M solution of (ani)_2_CH^+^ BF_4_^−^ (**4 e**) in CD_2_Cl_2_ at ambient temperature neither resulted in decolorization of the solution nor in a change of the ^1^H NMR spectrum of **4 e** within 10 minutes. After 1 h, about one‐third of **4 e** was reduced with formation of bis(4‐methoxyphenyl)methane, indicating that hydride transfer will disturb the kinetics of Lewis adduct formation with more reactive benzhydrylium ions. This observation is in line with Penczek's thorough investigation of the interactions of ethers and acetals with Ph_3_C^+^, which has an electrophilicity similar to **4 e**.^[9d]^ For the coordination of Ph_3_C^+^ with Et_2_O (**1**) at 25 °C an equilibrium constant *K*=0.23 M^−1^,^[14]^ and for the hydride transfer from Et_2_O to Ph_3_C^+^ a second‐order rate constant of 3×10^−4^ M^−1^ s^−1^ was determined.^[15]^

**Figure 1:**
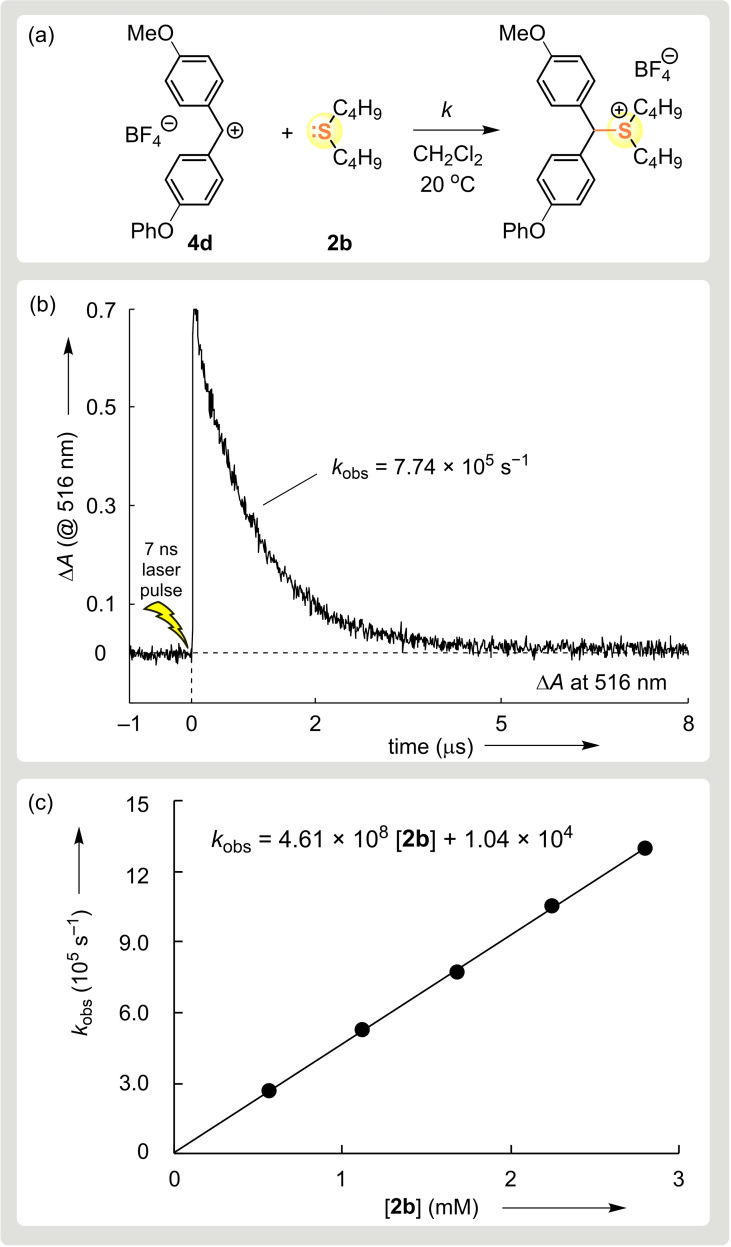
a) Kinetics of the reaction of **2 b** ([**2 b**]_0_=1.68 mM) with **4 d** at 20 °C in CH_2_Cl_2_ were monitored b) by the exponential decay of the absorbance of **4 d** at 516 nm during the course of the reaction (**4 d** generated by laser flash photolysis of **4 d**‐PPh_3_BF_4_). c) The second‐order rate constant *k*=4.61×10^8^ M^−1^ s^−1^ corresponds to the slope of the linear correlation of *k*_obs_ with [**2 b**].

All reactions of the benzhydrylium ions **4** with the thioethers **2 a**–**d** and dimethyl selenide **3** reported herein were well behaved and gave rise to mono‐exponential decays of the absorbances of **4** as shown in Figure 1 for the reaction of **4 d** with **2 b**. Because of the low concentrations of PPh_3_ generated by photolytic cleavage, recombination of PPh_3_ with the benzhydrylium ions did not compete with the reactions of the chalcogenides. First‐order rate constants *k*_obs_ [s^−1^] were obtained by fitting the mono‐exponential function *A*_t_=*A*_0_ *e* ${}^{-k{}_{\mathrm{obs}}t}$ +*C* to the decays of the absorbances. Plots of *k*_obs_ versus the concentrations of the nucleophiles were linear, indicating second‐order rate laws, and the second‐order rate constants *k* [M^−1^ s^−1^] listed in Table 2 were derived from the slopes of such plots.

**Table 2:** Second‐order rate constants (*k*) for the reactions of the thio‐ and selenoethers **2** and **3** with benzhydrylium ions **4** in CH_2_Cl_2_ at 20 °C.

Nucleophile	*N* / *s*_N_^[a]^	Ar_2_CH^+^		*k* [M^−1^ s^−1^]
Me_2_S (**2 a**)	12.32, 0.72	(fur)_2_CH^+^	**4 g**	8.10×10^7^
		(fur)(ani)CH^+^	**4 f**	2.02×10^8^
		(ani)_2_CH^+^	**4 e**	5.14×10^8 [b]^
		(ani)(pop)CH^+^	**4 d**	8.80×10^8 [b]^
**2 a** *in MeCN*	(12.7, 0.72)^[c]^	(fur)_2_CH^+^	**4 g**	1.55×10^8^
		(fur)(ani)CH^+^	**4 f**	3.29×10^8 [b]^
		(ani)_2_CH^+^	**4 e**	7.46×10^8 [b]^
		(ani)(pop)CH^+^	**4 d**	1.22×10^9 [b]^
		(ani)(Ph)CH^+^	**4 b**	2.87×10^9 [b]^
*n*Bu_2_S (**2 b**)	11.86, 0.74	(fur)_2_CH^+^	**4 g**	5.13×10^7^
		(fur)(ani)CH^+^	**4 f**	1.30×10^8^
		(ani)_2_CH^+^	**4 e**	2.73×10^8 [b]^
		(ani)(pop)CH^+^	**4 d**	4.61×10^8 [b]^
THT (**2 c**)	(13.1, 0.72)^[c]^	(fur)_2_CH^+^	**4 g**	2.88×10^8 [b]^
		(fur)(ani)CH^+^	**4 f**	3.85×10^8 [b]^
		(ani)_2_CH^+^	**4 e**	8.15×10^8 [b]^
		(ani)(pop)CH^+^	**4 d**	1.22×10^9 [b]^
**2 c** *in MeCN*	(13.3, 0.72)^[c]^	(fur)_2_CH^+^	**4 g**	3.99×10^8 [b]^
		(fur)(ani)CH^+^	**4 f**	6.89×10^8 [b]^
		(ani)_2_CH^+^	**4 e**	1.65×10^9 [b]^
		(ani)(pop)CH^+^	**4 d**	1.68×10^9 [b]^
		(ani)(Ph)CH^+^	**4 b**	4.97×10^9 [b]^
		(pop)(Ph)CH^+^	**4 a**	5.17×10^9 [b]^
THTP (**2 d**)	11.94, 0.75	(fur)_2_CH^+^	**4 g**	8.37×10^7^
		(fur)(ani)CH^+^	**4 f**	2.16×10^8^
		(ani)_2_CH^+^	**4 e**	5.57×10^8 [b]^
		(ani)(pop)CH^+^	**4 d**	7.34×10^8 [b]^
Me_2_Se (**3**)	(12.6, 0.72)^[c]^	(fur)(ani)CH^+^	**4 f**	3.16×10^8 [b]^
		(ani)_2_CH^+^	**4 e**	4.90×10^8 [b]^
		(ani)(pop)CH^+^	**4 d**	1.08×10^9 [b]^
		(ani)(tol)CH^+^	**4 c**	2.06×10^9 [b]^

[a] Nucleophile‐specific parameters *N* and *s*_N_ according to Equation (1). [b] Because of the proximity of the diffusion limit, not used for the calculation of *N* and *s*_N_. [c] As the available rate constants are close to the diffusion limit, they are not used for the calculation of *N* and *s*_N_; for that reason, *s*_N_=0.72 was assumed to be the same as for structurally analogous nucleophiles and combined with the smallest rate constant of the series to obtain an estimate for *N*.

### Determination of nucleophile‐specific parameters *N* and *s*_N_

As previously reported for numerous reactions of benzhydrylium ions with different families of nucleophiles, plots of log *k* versus electrophilicity *E* are generally linear up to rate constants of approximately 2×10^8^ M^−1^ s^−1^ and flatten as the diffusion limit is approached.^[11]^ The linear parts of these correlations, which follow Equation (1), have been used to derive *N* from the intercepts on the abscissa and *s*_N_ as the slopes.^[9]^

**Figure 2:**
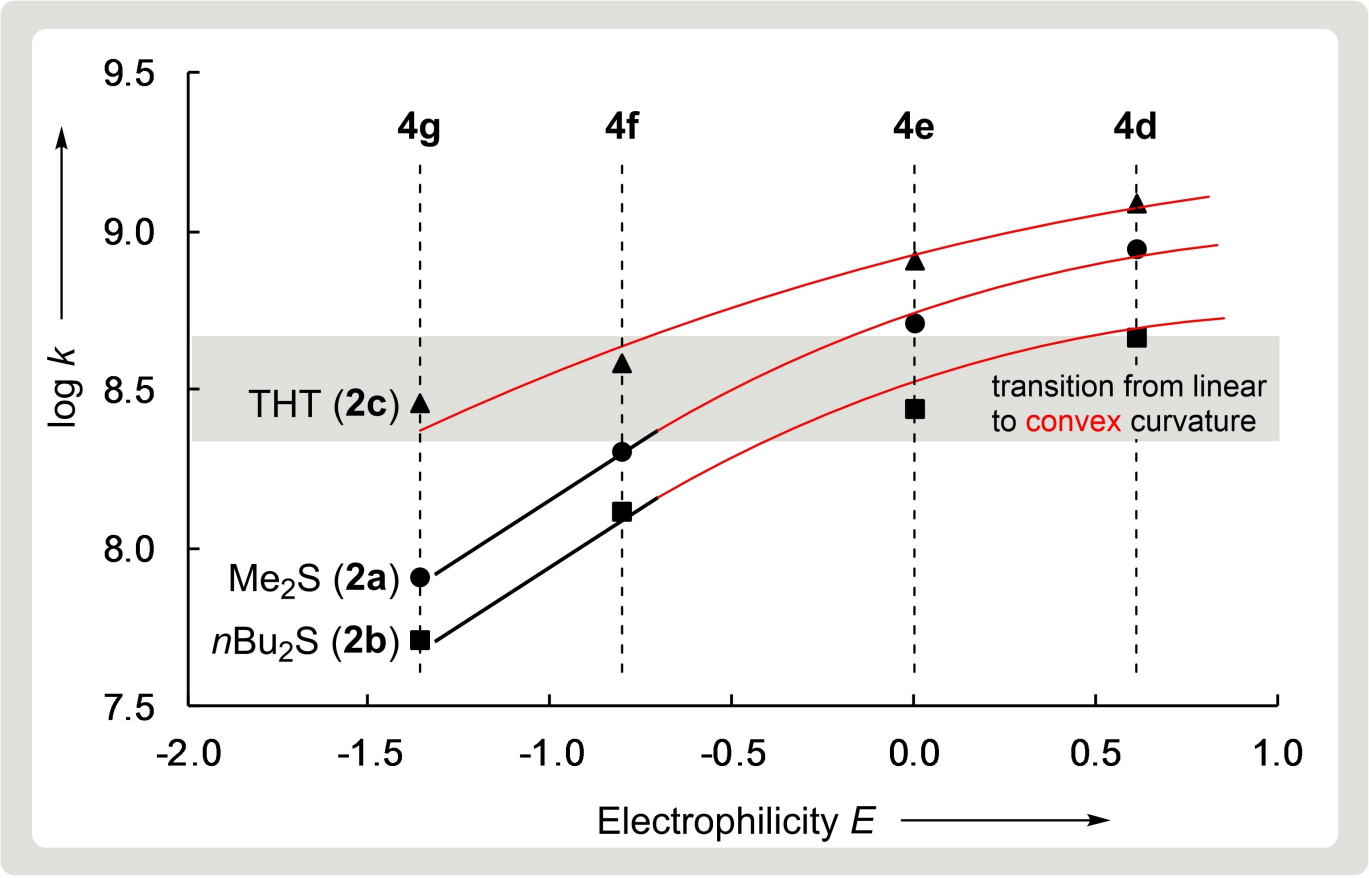
Plot of log *k* versus the electrophilicity parameter *E* for the reactions of dimethyl sulfide (**2 a**), dibutyl sulfide (**2 b**), and tetrahydrothiophene (THT, **2 c**) with benzhydrylium ions **4** in CH_2_Cl_2_ at 20 °C.

Analogously, Figure 2 shows a flattening of log *k* versus *E* plots when the second‐order rate constants *k* for the reactions of dialkyl sulfides **2** with benzhydrylium ions **4** exceed 2×10^8^ M^−1^ s^−1^. Only the reactions of the least electrophilic benzhydrylium ions **4 f** and **4 g** with the thioethers **2 a**, **2 b**, and **2 d** proceed with rate constants smaller than 2×10^8^ M^−1^ s^−1^ and can be used to evaluate *N* and *s*_N_ for these nucleophiles in the conventional way as illustrated in Figure 2. Since all rate constants determined for the stronger nucleophiles **2 c** and **3** are greater than 2×10^8^ M^−1^ s^−1^, a different way for characterizing the nucleophilic reactivities of **2 c** and **3** was needed.

Reactions with less electrophilic carbocations cannot be used for this purpose because such carbocations do not give adducts at 20 °C due to the lack of a thermodynamic driving force. As specified in the footnotes of Table 2, approximate *N* values for the more nucleophilic Lewis bases **2 c** and **3** (and for **2 a** in acetonitrile) were calculated from the rate constants of the slowest reactions of these chalcogenides by applying Equation (1) with an assumed *s*_N_=0.72, the susceptibility determined for the structurally related dimethyl sulfide **2 a**. Similar values of *s*_N_ have also been obtained for **2 b** and **2 d**.

### Intrinsic barriers

For some of the reactions listed in Table 2, equilibrium constants have previously been determined^[10a]^ (Table 3). According to the Marcus Equation (2), the activation energy Δ*G* ^≠^ of a reaction can be expressed by the combination of the reaction Gibbs energy Δ_r_ *G*° with the intrinsic barrier Δ*G*_0_^≠^, which corresponds to the activation Gibbs energy for a reaction with Δ_r_ *G*°=0., ^[16]^

(2) $$\Delta G{}^{\neq }=\Delta G{}_{0}{}^{\neq }+0.5\;\Delta {}_{r}G{}^{\circ }+\left(\Delta {}_{r}G{}^{\circ }\right){}^{2}/\left(16\;\Delta G{}_{0}{}^{\neq }\right)$$

**Table 3:** Equilibrium constants *K*, Gibbs reaction energies Δ_r_ *G*_0_, Gibbs activation energies Δ*G* ^≠^, and Marcus intrinsic barriers Δ*G*_0_^≠^ for the reactions of the thio‐ and selenoethers **2** and **3** with benzhydrylium ions (Ar_2_CH^+^, **4**) in CH_2_Cl_2_ at 20 °C.

Nucleophile (Lewis base)	Ar_2_CH^+^	**4**	*K*^[a]^ [M^−1^]	Δ_r_ *G*°^[a]^ [kJ mol^−1^]	Δ*G* ^≠[b]^ [kJ mol^−1^]	Δ*G*_0_^≠[c]^ [kJ mol^−1^]
Me_2_S (**2 a**)	(fur)_2_CH^+^	**4 g**	2.16×10^2^	−13.1	27.4	33.6
	(ani)_2_CH^+^	**4 e**	8.91×10^3^	−22.2	22.9	33.0
	(ani)(pop)CH^+^	**4 d**	6.67×10^4^	−27.1	21.6	33.7
*n*Bu_2_S (**2 b**)	(fur)_2_CH^+^	**4 g**	1.52×10^2^	−12.2	28.5	34.3
	(ani)_2_CH^+^	**4 e**	6.15×10^3^	−21.3	24.4	34.2
	(ani)(pop)CH^+^	**4 d**	3.61×10^4^	−25.6	23.1	34.7
THT (**2 c**)	(fur)_2_CH^+^	**4 g**	7.94×10^2^	−16.3	24.3	31.9
	(ani)_2_CH^+^	**4 e**	1.22×10^4^	−22.9	21.7	32.2
	(ani)(pop)CH^+^	**4 d**	6.43×10^4^	−27.0	20.8	32.9
THTP (**2 d**)	(fur)_2_CH^+^	**4 g**	8.86×10^2^	−16.5	27.3	35.1
	(ani)_2_CH^+^	**4 e**	3.04×10^4^	−25.2	22.7	34.1
	(ani)(pop)CH^+^	**4 d**	1.05×10^5^	−28.2	22.0	34.7
Me_2_Se (**3**)	(ani)_2_CH^+^	**4 e**	9.50×10^2^	−16.7	23.0	30.8
	(ani)(pop)CH^+^	**4 d**	7.65×10^3^	−21.8	21.0	31.0

[a] From ref. [10a]. [b] Calculated by applying the Eyring equation on the rate constants in Table 2. [c] Calculated by using Marcus Equation (2).

Substitution of Δ_r_ *G*° and Δ*G* ^≠^ into Equation (2) yields the intrinsic barriers Δ*G*_0_^≠^. Values of Δ*G*_0_^≠^ determined for the reactions of nucleophiles **2** and **3** with benzhydrylium ions (**4**) are listed in Table 3. Zhu has criticized Marcus′ derivation of Equation (2) and developed an alternative expression, which differs from Equation (2) by the absence of the last term.^[17]^ Since the intrinsic barriers calculated by Zhu′s equation differ only insignificantly from those calculated by Equation (2) (+0.4 to 1.4 kJ mol^−1^), we only list the Marcus intrinsic barriers in Table 3 in order to retain comparability with the majority of published intrinsic barriers.

In previous work, we showed that within a reaction series (one nucleophile with different benzhydrylium ions) Marcus intrinsic barriers Δ*G*_0_^≠^ decrease with increasing reactivity of the benzhydrylium ions for nucleophiles with *s*_N_>0.67, and increase for nucleophiles with *s*_N_<0.67.^[18]^ Accordingly, the intrinsic barriers are almost constant within the different reaction series of Table 3, because the corresponding susceptibility parameters (0.72<*s*_N_<0.75) are close to 0.67.

Intrinsic barriers below 35 kJ mol^−1^, as derived for the reactions of benzhydrylium ions with thio‐ and selenoethers (Table 3), are the smallest ones we have observed so far. These low intrinsic barriers account for the fact that only a small number of benzhydrylium ions undergo activation‐controlled reactions with **2** and **3**. The reactions of **2** and **3** with slightly more reactive carbenium ions occur under diffusion control, while the reactions of **2** and **3** with slightly less reactive carbenium ions do not occur at all (i. e., no products are formed).

Intrinsic barriers Δ*G*_0_^≠^, that is, the barriers for reactions with reaction Gibbs energies Δ_r_ *G*°=0, are linked to the reorganization energies λ by the relationship Δ*G*_0_^≠^=*λ*/4. Since little structural reorganization is required when electrophiles attack at the lone electron pair of sp^3^‐hybridized atoms, dialkyl chalcogenides as well as tertiary amines (for DABCO and quinuclidine, Δ*G*_0_^≠^≈40 kJ mol^−1^)^[10b]^ react via low intrinsic barriers. In previous work, the kinetically controlled S attack at thiocyanate ions has also been assigned to the lower reorganization energy for this site of attack (Δ*G*_0_^≠^=35–38 kJ mol^−1^ for the S attack at SCN^−^).^[19]^

### Nucleofugalities

In analogy to Equation (1), Equation (3) has been used for the construction of a comprehensive nucleofugality scale,^[12]^ which allows one to calculate the rates of heterolytic cleavages *k*_rev_ [s^−1^] of R−X from the electrofugality parameter *E*_f_ of R^+^ and the solvent‐specific nucleofuge parameters *N*_f_ and *s*_f_ of X or X^−^.

(3) $$\log\;k{}_{\mathrm{rev}}\left(25\;{}^{\circ }C\right)=s{}_{f}\left(E{}_{f}+N{}_{f}\right)$$

**Figure 3:**
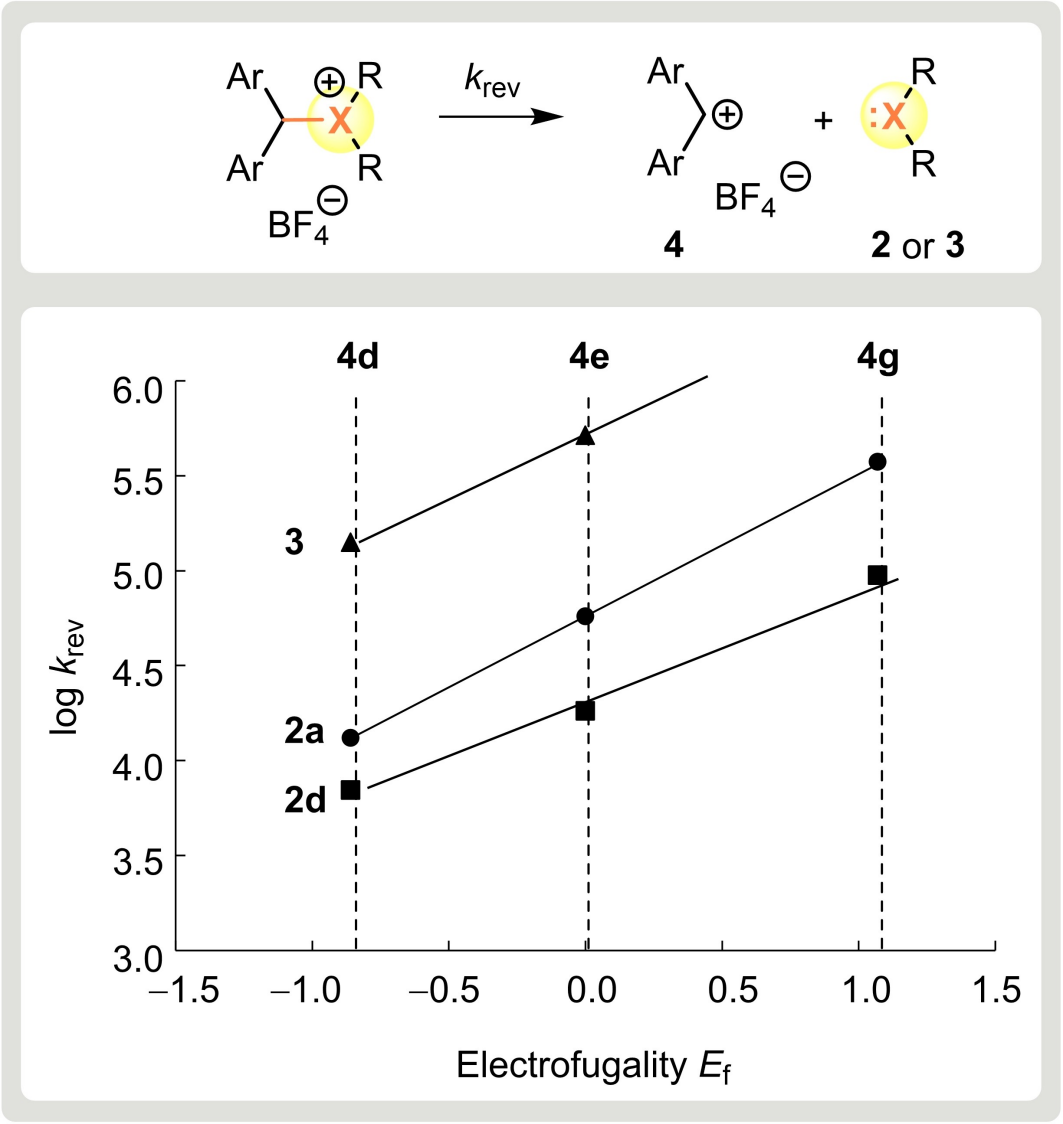
Plot of the reverse rate constants (log *k*_rev_) for the reactions of nucleophiles **2** and **3** with benzhydrylium ions **4** in CH_2_Cl_2_ at 20 °C against the electrofugality parameters *E*_f_ of **4** (see the Supporting Information for analogous log *k*_rev_ vs. *E*_f_ plots for **2 b** and **2 c**).

From the rate constants *k* of the reactions of the benzhydrylium ions **4** with the thio‐ and selenoethers **2** and **3** determined in this work (Table 2) and the previously reported equilibrium constants *K*,^[10a]^ the heterolysis rate constants *k*_rev_ [s^−1^] of the corresponding trialkyl sulfonium ions have now been calculated as the ratios *k*/*K* at 20 °C in CH_2_Cl_2_ (Table 4, column 5). Plots of log *k*_rev_ against the known electrofugality parameters *E*_f_ of benzhydrylium ions,^[12]^ resulted in linear correlations (Figure 3) from which the nucleofuge‐specific parameters *s*_f_ and *N*_f_ for **2** and **3** were derived (Table 4).

**Table 4:** Reverse rate constants (*k*_rev_) for the reactions of nucleophiles **2** and **3** with benzhydrylium ions (Ar_2_CH^+^, **4**) and the resulting nucleofuge‐specific parameters (*N*_f_ and *s*_f_) in CH_2_Cl_2_ at 20 °C.

Nucleofuge	*N*_f_, *s*_f_^[a]^	Ar_2_CH^+^		*E*_f_^[b]^	*k*_rev_^[c]^ [s^−1^]	*k*_rev_^[d]^ [s^−1^]
Me_2_S (**2 a**)	6.33, 0.75	(fur)_2_CH^+^	**4 g**	1.07	3.75×10^5^	1.04×10^3^
		(ani)_2_CH^+^	**4 e**	0.00	5.77×10^4^	1.25×10^2^
		(ani)(pop)CH^+^	**4 d**	−0.86	1.32×10^4^	2.28×10^1^
*n*Bu_2_S (**2 b**)	6.36, 0.74	(fur)_2_CH^+^	**4 g**	1.07	3.38×10^5^	
		(ani)_2_CH^+^	**4 e**	0.00	4.44×10^4^	
		(ani)(pop)CH^+^	**4 d**	−0.86	1.28×10^4^	
THT (**2 c**)	7.26, 0.66	(fur)_2_CH^+^	**4 g**	1.07	3.63×10^5^	1.38×10^3^
		(ani)_2_CH^+^	**4 e**	0.00	6.68×10^4^	1.66×10^2^
		(ani)(pop)CH^+^	**4 d**	−0.86	1.90×10^4^	3.01×10^1^
THTP (**2 d**)	7.33, 0.59	(fur)_2_CH^+^	**4 g**	1.07	9.45×10^4^	
		(ani)_2_CH^+^	**4 e**	0.00	1.83×10^4^	
		(ani)(pop)CH^+^	**4 d**	−0.86	6.99×10^3^	
Me_2_Se (**3**)	8.72, 0.66	(ani)_2_CH^+^	**4 e**	0.00	5.16×10^5^	
		(ani)(pop)CH^+^	**4 d**	−0.86	1.41×10^5^

[a] From Equation (3). [b] From ref. [12]. [c] Calculated by the relation *k*_rev_=*k*/*K* in Tables 2 and 3. [d] Extrapolated by Equation (3) from *E*_f_ in Table 4 and solvolysis rate constants in ethanol at 25 °C (from ref. [20]).

Jurić, Denegri, and Kronja recently used Equation (3) to derive the nucleofugalities of dimethyl sulfide (**2 a**) and THT (**2 c**) from the solvolysis rate constants *k*_rev_ [s^−1^] of the corresponding benzhydryl sulfonium ions in pure and aqueous ethanol and methanol.[^20^, ^21^] By using their *N*_f_ and *s*_f_ parameters[^20^, ^21^] to calculate *k*_rev_ for the corresponding sulfonium ions in ethanol at 25 °C by Equation (3) one arrives at values for *k*_rev_ that are 2 to 3 orders of magnitude smaller than those calculated from the *k*/*K* ratios in dichloromethane at 20 °C (two right columns of Table 4). This large difference cannot be due to the use of different solvents used in both studies because a variation of the solvent has been reported to have only a minor effect on the nucleofugality of neutral leaving groups.[^20^, ^21^, ^22^]

**Figure 4:**
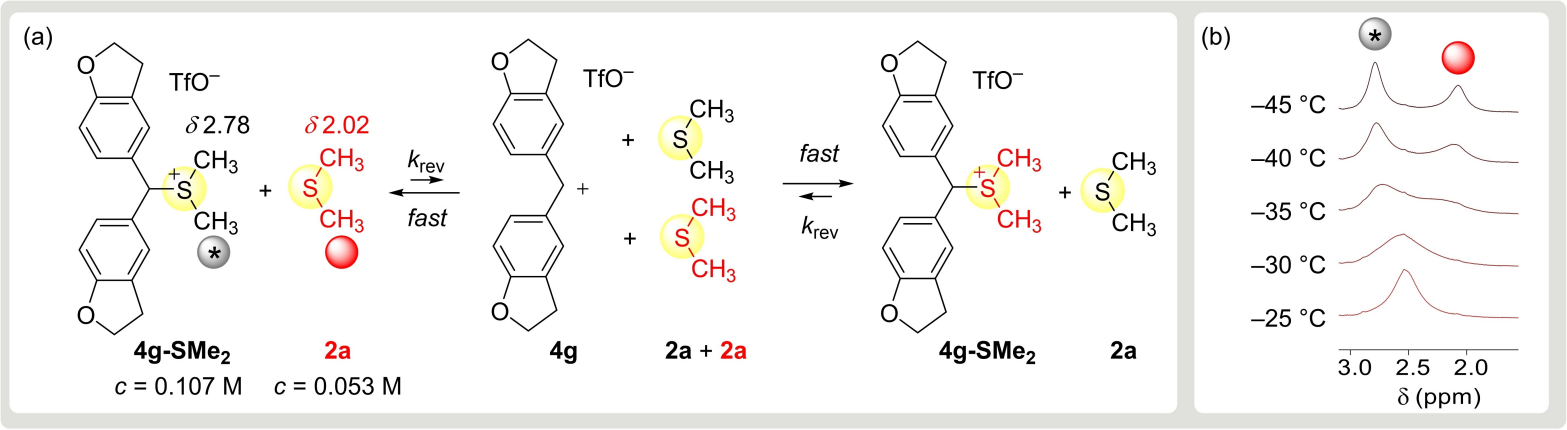
a) Dimethylsulfide exchange between **4 g**‐SMe_2_ and free **2 a** in CD_2_Cl_2_. b) Temperature‐dependent proton resonances for the *S*‐methyl groups evaluated by line‐shape analysis (DNMR6 algorithm) for the determination of exchange rate constants *k*_rev_(*T*).

In order to resolve the discrepancy of *k*_rev_ determined in this and earlier work[^20^, ^21^] we performed dynamic NMR (DNMR) studies in analogy to previously reported DNMR investigations of trialkyl sulfonium ions.^[23]^ The *S*‐methyl groups of a mixture of **4 g**‐SMe_2_ and Me_2_S (**2 a**, marked red in Figure 4) in CD_2_Cl_2_ at −80 °C resonated separately at *δ* 2.78 for **4 g**‐SMe_2_ and *δ* 2.02 for **2 a** (red) in the 400 MHz ^1^H NMR spectrum (Figure 4). Coalescence of these *S*‐methyl resonances was observed when the temperature was gradually raised (Figure 4b). Because the concentration of Me_2_S arising from heterolytic cleavage of the sulfonium ion **4 g**‐SMe_2_ (black in Figure 4) is low compared to the concentration of extra Me_2_S in solution (red in Figure 4), benzhydrylium ion **4 g** reacts preferentially with **2 a** from the solution and gives rise to the observed dynamic NMR phenomenon. Line shape analysis (LSA) for ^1^H NMR spectra in the temperature range from −45 to −25 °C furnished the corresponding exchange rate constants *k*_rev_(*T*) and Eyring activation parameters (Supporting Information), which allowed us to extrapolate a first‐order rate constant of 1.3×10^5^ s^−1^ for the Me_2_S exchange at 20 °C. Non‐coordinated **4 g** is not detectable in the NMR spectrum, in accord with the equilibrium constant *K*=216 M^−1^ for the Lewis adduct formation from **4 g** and **2 a** (Table 3). The (pseudo‐)first‐order rate constant for the reaction of **4 g** with **2 a** can be calculated as 4.3×10^6^ s^−1^ (=8.1×10^7^ M^−1^ s^−1^×0.053 M) under these conditions. Since this value is 33 times greater than the exchange rate constant determined by DNMR, we can conclude that the latter (*k*_rev_ in Figure 4) corresponds to the heterolytic cleavage of the carbon‐sulfur bond in the sulfonium ion **4 g**‐SMe_2_.

The value of *k*_rev_ for **4 g**‐SMe_2_ determined by DNMR is three times smaller than that derived as *k*/*K* from experimentally measured equilibrium and forward rate constants (*k*_rev_ in CH_2_Cl_2_, Table 4), an acceptable agreement in view of the completely different ways of derivation. The analogous DNMR investigation of (ani)_2_CH‐SMe_2_^+^ (**4 e**‐SMe_2_) in CD_2_Cl_2_ in the temperature range from −50 to 0 °C resulted in an extrapolated *k*_rev_(20 °C)=2.6×10^4^ s^−1^, two times smaller than *k*_rev_ derived from the *k*/*K* ratio.

After confirming the correct order of magnitude of *k*_rev_ derived from *k*/*K* for alkoxy‐substituted benzhydryldimethylsulfonium ions, let us return to the question, why the *k*_rev_ values in Table 4, column 5 are so much larger than those calculated by Equation (3) from the ethanolysis rate constants of the Zagreb group (Table 4, column 6).

**Figure 5:**
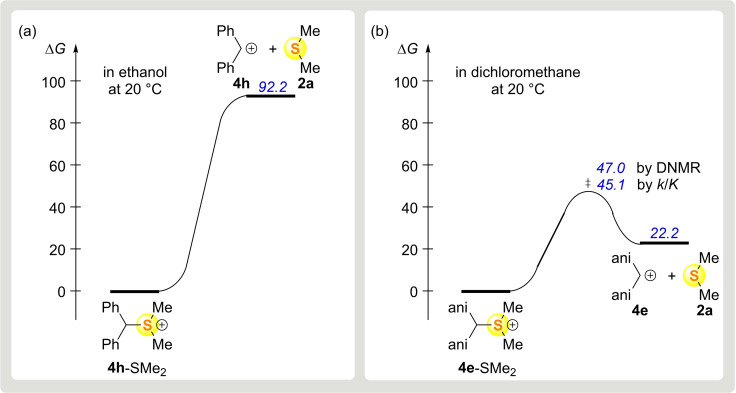
Gibbs energy [kJ mol^−1^] profiles for the heterolysis of benzhydryldimethylsulfonium ions a) **4 h**‐SMe_2_ and b) **4 e**‐SMe_2_.

The ethanolysis rate constants in refs[^20^, ^21^] refer to benzhydryldimethylsulfonium ions composed of the parent and halogen‐substituted benzhydrylium ions, all of which are highly electrophilic (*E*≥5.20).^[9]^ Substitution of this *E* value and the nucleophilicity parameters of **2 a** and **2 c** (Table 2) into Equation (1) shows that all benzhydrylium ions generated in the ethanolysis studies undergo diffusion‐controlled reactions with the thioethers **2** (*k* ^eq(1)^>10^10^ M^−1^ s^−1^). In contrast, the reactions of the thioethers **2** with the alkoxy‐substituted benzhydrylium ions **4 d**–**g** investigated in this work are activation controlled (Table 2). As a consequence of the principle of microscopic reversibility, the transition states of the heterolyses of the parent and halogen‐substituted benzhydryl sulfonium ions *correspond to* the carbocations (Figure 5a), while those of the alkoxy‐substituted analogues only *resemble* the carbocations (Figure 5b).

As linear free energy relationships break down when a change from activation‐control to diffusion‐control[^10b^, ^10c^] is involved, one can explain why the nucleofuge‐specific parameters *N*_f_ and *s*_f_ derived from ethanolysis rate constants (Figure 5a) are not applicable to the heterolysis rates of the alkoxy‐substituted benzhydryl sulfonium ions (Figure 5b).

### Relationships between structures and reactivities

The observation that diethyl ether (**1**) does not form adducts (*K*<10) with benzhydrylium ions of *LA*<1 (Table 1) at 20 °C shows that ethers are weaker Lewis bases towards carbenium ions than structurally related sulfides. This ordering is in analogy with the relative proton affinities in the gas phase (Me_2_S 197, Me_2_O 186 kcal mol^−1^), but not with the relative Brønsted basicities in an aqueous solution [p*K*_aH_(Me_2_O)=−2.52 vs. p*K*_aH_(Me_2_S)=−6.95].^[6]^

**Scheme 4:**
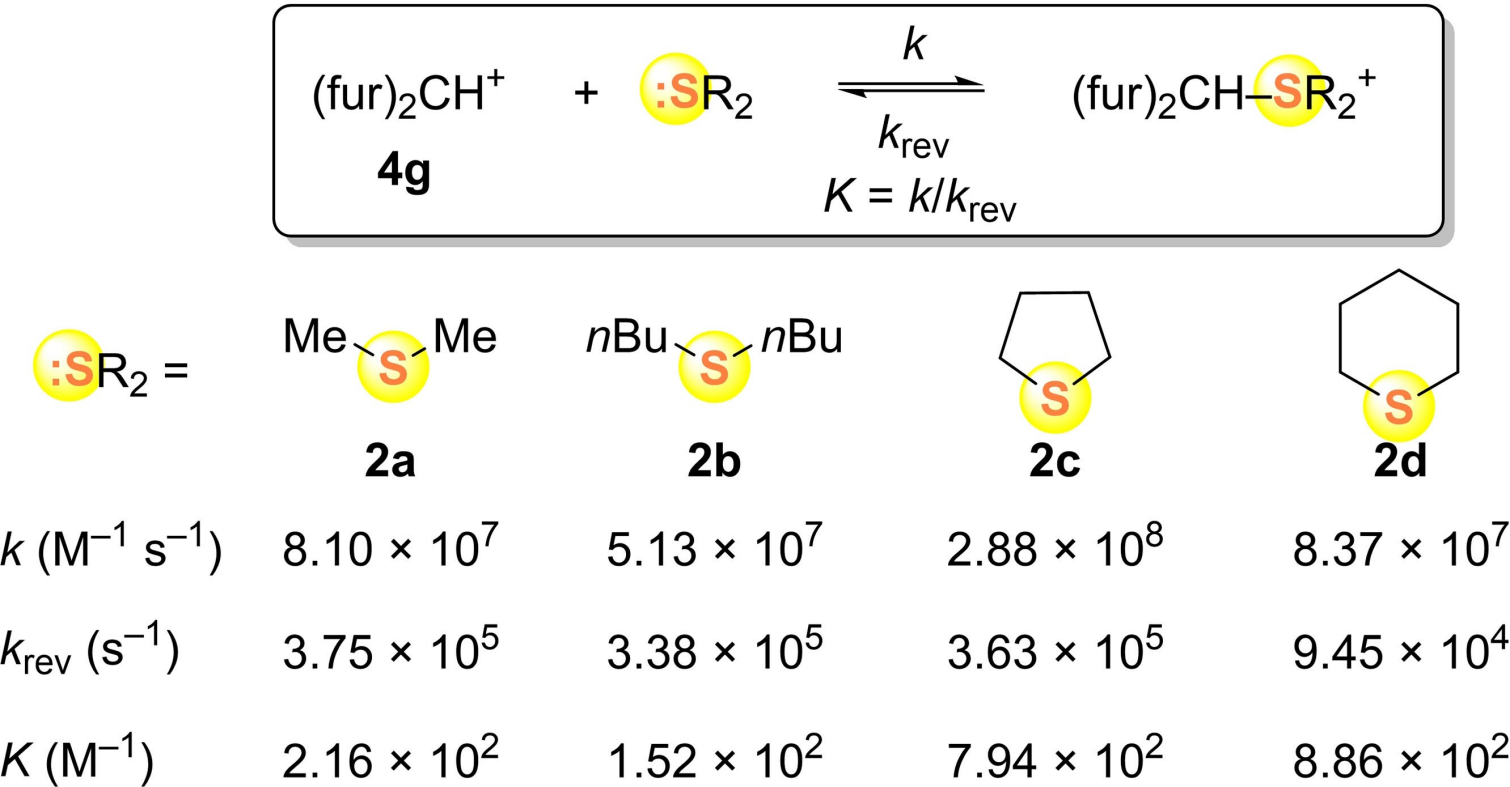
Rate and equilibrium constants for the reactions of thioethers **2** with the benzhydrylium ion **4 g** in CH_2_Cl_2_ at 20 °C (with *k*_rev_=*k*/*K*).

Scheme 4 shows that Me_2_S (**2 a**) is a slightly stronger nucleophile and stronger Lewis base than *n*Bu_2_S (**2 b**), with the consequence that the nucleofugalities (*k*_rev_) of these two sulfides are almost identical. Interestingly, dimethyl sulfide (**2 a**) and tetrahydrothiopyran (**2 d**) have the same nucleophilic reactivity, though the cyclic sulfide **2 d** is a four‐times stronger Lewis base. The higher intrinsic barriers in reactions of **2 d** (Table 3), which account for this ranking, also account for the fact that despite the comparable Lewis basicities of the cyclic sulfides **2 c** and **2 d**, tetrahydrothiopyran (**2 d**) reacts 3‐times more slowly than tetrahydrothiophene (**2 c**) with **4 g**.

**Scheme 5:**
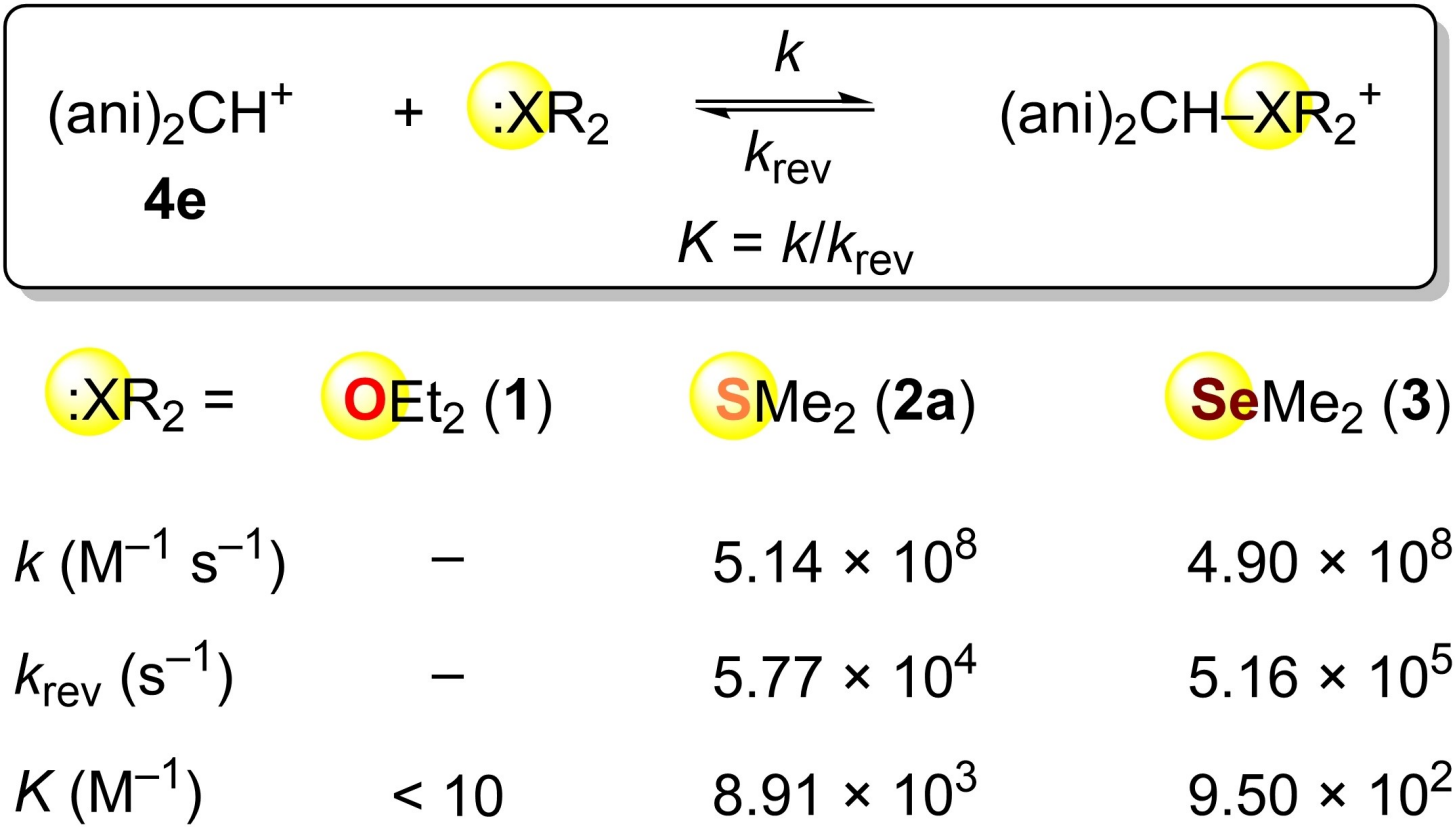
Rate and equilibrium constants for the reactions of dialkyl chalcogenides with the benzhydrylium ion **4 e** in CH_2_Cl_2_ at 20 °C (with *k*_rev_=*k*/*K*).

Due to the low Lewis basicity of dimethylselenide (**3**), its reaction with (fur)_2_CH^+^ (**4 g**) proceeds only with a very low degree of conversion. For that reason, its nucleophilic reactivity could not be included in the comparisons of Scheme 4. On the other hand, Scheme 5 shows that the more Lewis acidic benzhydrylium ion **4 e** can be used as a reference for the comparison of dimethyl selenide (**3**) with dimethyl sulfide (**2 a**).

Though dimethyl sulfide (**2 a**) is a tenfold stronger Lewis base towards benzhydrylium ions than dimethyl selenide (**3**), according to Table 3, both compounds have equal nucleophilicities, indicating a higher intrinsic barrier for the reaction of **2 a**. As a consequence, selenoether **3** is a tenfold better nucleofuge than thioether **2 a** (Scheme 5).

## Conclusion

**Figure 6:**
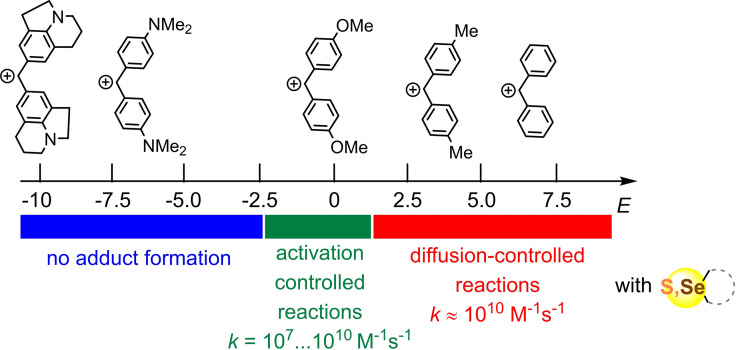
Scope of reactions of thio‐ and selenoethers with benzhydrylium ions of different electrophilicity *E*.

Dialkyl sulfides **2 a**–**d** and dimethyl selenide **3** react with benzhydrylium ions (which can be considered representative carbenium ions and π‐electrophiles in general) with unusually low intrinsic barriers. For this reason, these thio‐ and selenoethers undergo diffusion‐controlled reactions with almost all carbenium ions that have sufficiently high Lewis acidities for the reactions to be exergonic. Thus, only a small number of carbenium ions undergo activation‐controlled reactions with these dialkyl chalcogenides, providing rate constants that can be used to calculate the corresponding nucleophilicity parameters *N* and *s*_N_ from Equation 1 (Figure 6).

**Figure 7:**
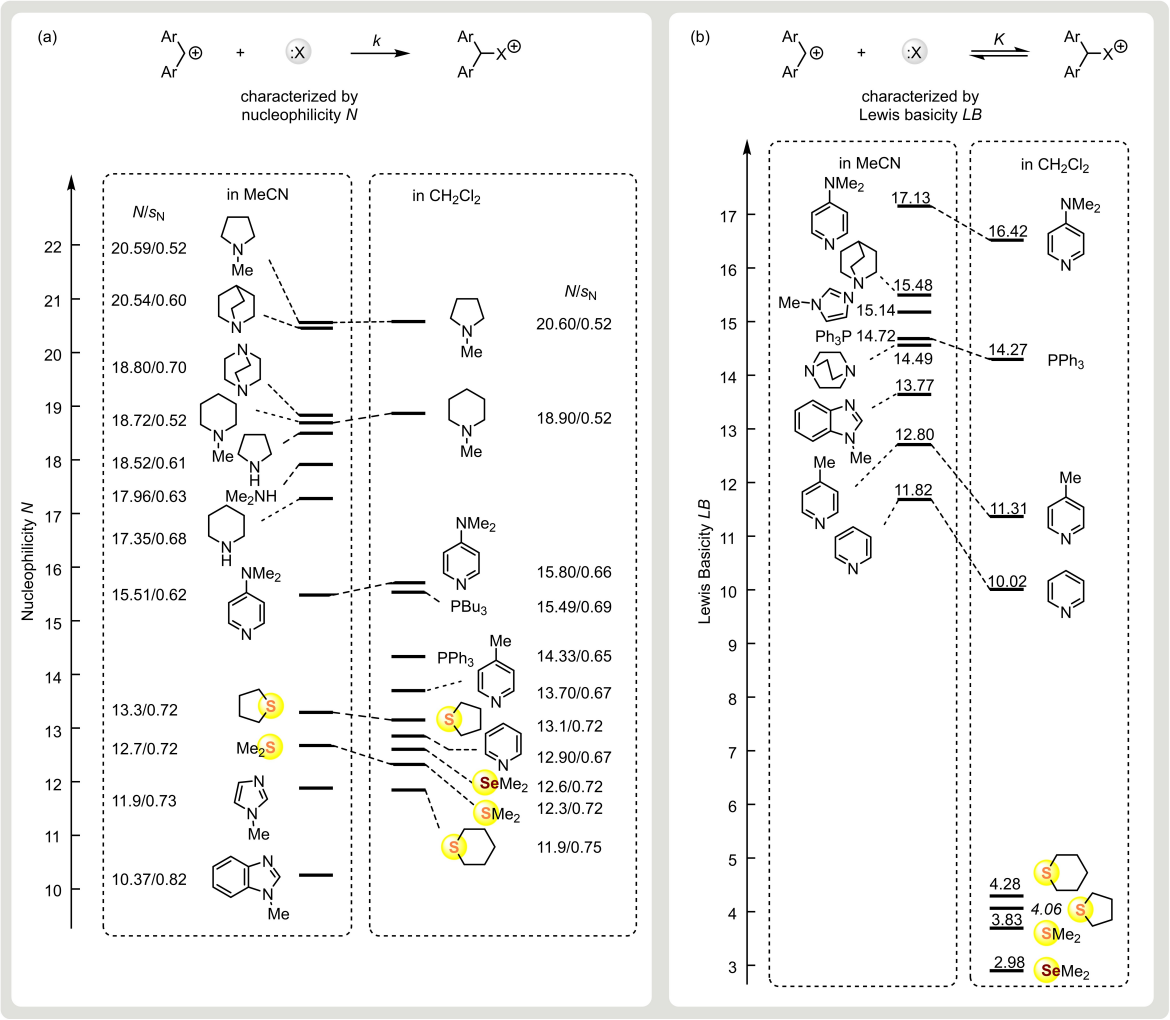
a) Nucleophilicity parameters *N*/*s*_N_ and b) Lewis‐basicity parameters *LB* of chalcogenides and pnictogenides in acetonitrile and dichloromethane.

Because of the similarities of their susceptibilities *s*_N_, the relative reactivities of the nucleophiles depicted in Figure 7 exhibit only a minor dependence on the nature of their electrophilic reaction partners. For that reason, their relative nucleophilic reactivities can be approximated by the nucleophilicity parameters *N*. Several entries in Figure 7 show that the nucleophilicities *N* and Lewis basicities *LB* depend only slightly on the solvent (CH_2_Cl_2_ vs. MeCN) due to the fact that the combination of a cation and a neutral reactant yields another cation. For that reason, we shall not specifically mention solvent effects in the following discussion.

The thioethers **2** and the selenoether **3** cover a very small range of both the nucleophilicity and the Lewis basicity scales in Figure 7. Although they are by far the weakest Lewis bases in this ranking, their nucleophilicities are comparable to those of several commonly used organocatalysts. Thus, dialkyl chalcogenides **2** and **3** have a similar nucleophilicity to pyridine and *N*‐methylimidazole, though the Lewis basicities of the latter derived from equilibrium constants of their reactions with benzhydrylium ions are 8 to 11 orders of magnitude greater. Analogously, the nucleophilic reactivities of the chalcogenides are only 10 to 1000 times smaller than those of PPh_3_ and PBu_3_, whereas the corresponding Lewis basicities differ by more than 10 orders of magnitude. Both comparisons reflect the very low intrinsic barriers of the reactions of the chalcogenides.

What is the reason for the low Marcus intrinsic barriers of these reactions of chalcogenides which are responsible for their unique reactivities? Hoz has reported that the barriers of the identity reactions in Equation (4) (i. e., the intrinsic barriers) decrease, the further right X is in the periodic table,^[24]^ which can be explained by increasing electronegativity of X.

(4) $$X{}^{-}+H{}_{3}C-X\to X-\mathrm{CH}{}_{3}+X{}^{-}$$

That the intrinsic barriers remain almost constant as one goes down a particular group in the periodic table was explained by Arnaut and colleagues by decreasing C−X force constants associated with flattening of the intersecting parabola in the Marcus model,^[25a]^ which is almost compensated for by increasing the C−X bond length. More recent work showed that in analogous identity reactions with neutral nucleophiles at benzyl derivatives, intrinsic barriers increase as one goes down the periodic table, but again, the intrinsic barrier for SMe_2_ exchange is lower than that for NMe_3_ exchange.^[25b]^ As the relative nucleophilicities toward C_sp2_ centres have been reported to be linearly correlated with relative nucleophilicities toward C_sp3_ centres,^[26]^ the same reason appears to account for the fact that the intrinsic barriers for the reactions of S‐nucleophiles with carbenium ions are lower than those for N‐ and P‐nucleophiles.

The astonishing result that dialkyl sulfides and selenides are as nucleophilic as the much stronger (Brønsted and Lewis) bases pyridine and imidazole is not only due to the exceptionally low intrinsic barriers for the reactions of the chalcogenides but also due to the particularly high intrinsic barriers for the reactions of the N‐heteroarenes. As intrinsic barriers are associated with the degree of reorganization,[^16^, ^27^] the nuclear movements accompanying the reorganization of the aromatic π‐system during the electrophilic attack at the nitrogen of the heteroarenes^[28]^ account for the fact that pyridines and imidazoles react via higher intrinsic barriers than alkylamines with the consequence that N‐heteroarenes and alkylamines differ much more in nucleophilicity than in basicity (Figure 7).

The exceptionally low intrinsic barriers of the reactions of dialkyl sulfides and selenides, which are responsible for their high nucleophilicities despite their low Lewis basicities, also account for their high nucleofugalities and thus for their suitability as group‐transfer reagents.

## Conflict of interest

The authors declare no conflict of interest.

## Supporting information

As a service to our authors and readers, this journal provides supporting information supplied by the authors. Such materials are peer reviewed and may be re‐organized for online delivery, but are not copy‐edited or typeset. Technical support issues arising from supporting information (other than missing files) should be addressed to the authors.

Supporting Information
