# Supplementary material for: Nucleophilicities and Nucleofugalities of Thio‐ and Selenoethers
Source: Chemistry. 2021 Jun 30;27(44):11367–76. doi: 10.1002/chem.202100977 (PMC8456842; doi:10.1002/chem.202100977)
Supplement: Supplementary file 1 — Supporting Information [file CHEM-27-11367-s001.pdf]

Chemistry–A European Journal

Supporting Information

Nucleophilicities and Nucleofugalities of Thio- and Selenoethers

Biplab Maji, Xin-Hua Duan, Patrick M. Jüstel, Peter A. Byrne, Armin R. Ofial, and Herbert Mayr*

– Supporting Information –

Table of Contents

1. General Information and Methods S2
2. Product Characterization S3
3. Kinetics of the reactions of chalcogenides with benzhydrylium ions S5
4. Determination of nucleofugality parameters N_f and s_f for **2** and **3** S16
5. Dynamic ^{1}H NMR Spectroscopy and Line Shape Analysis S17
6. Copies of ^{1}H and ^{13}C NMR spectra of Lewis adducts S21
7. References.. S25

1. General Information and Methods

Chemicals. Dichloromethane was freshly distilled over CaH_2. Chalcogenides were purchased from commercial sources and freshly distilled before use. Trimethylsilyl triflate (>98%) was used as purchased. Benzhydryltriphenylphosphonium tetrafluoroborates were prepared as described before.[S1] Benzhydryl chlorides **4e**-Cl and **4g**-Cl were obtained from the reactions of benzhydrols with thionyl chloride in dichloromethane by following published procedures.[S2]

Analytics. A 400 MHz nuclear magnetic resonance (NMR) spectrometer was used to acquire ^{1}H and ^{13}C NMR spectra. ^{13}C NMR were recorded with broad-band proton decoupling. Abbreviations for NMR-data: s = singlet, d = doublet, t = triplet, quint = quintet, m = multiplet, br = broad. Chemical shifts are denoted as parts per million (ppm). NMR spectra were internally referenced to the residual signals of CD_2Cl_2 (δ_H = 5.32 ppm, δ_C = 54.00 ppm).[S3] Signals were assigned on the basis of additional HSQC, HMBC, and ^{1}H,^{1}H-COSY experiments.

Kinetics. The reactions of the dialkyl sulfides **2** and dimethyl selenide **3** with the colored benzhydrylium ions (Ar_2CH^+) were followed photometrically at or close to the absorption maxima of (Ar_2CH^+) by UV-vis spectroscopy as described previously.[S4] Reactions were analyzed by laser flash photolytic generation of benzhydrylium ions (Ar_2CH^+) from phosphonium ion precursor salts (**4**-PPh_3BF_4) in presence of excess **2** and **3**. A solution of known concentration of **4**-PPh_3BF_4 in CH_2Cl_2 (≈ 10^{-5} M) was mixed with a solution of known concentration of **2** and **3** (≈ 10^{-4} to 10^{-3} M) in CH_2Cl_2. The resulting colorless solution was then irradiated with 7-ns laser pulses (266 nm) from a quadrupled Nd:YAG laser (266 nm, 40-60 mJ/pulse) to generate the benzhydrylium ions Ar_2CH^+. The temperature of solutions was kept constant at (20.0 ± 0.1) °C during all kinetic studies by using a circulating bath thermostat. The pseudo-first-order rate constants k_{obs} (s^{-1}) were obtained by least-squares fitting of the mono-exponential function $A_t = A_0 \exp(-k_{obs}t) + C$ to the observed absorbances. The second-order rate constants k (M^{-1} s^{-1}) were obtained from the slopes of the linear plots of k_{obs} against the

concentrations of the nucleophiles. Tables with concentrations of reactants and individual rate constants are collected in the Supporting Information.

Dynamic NMR Spectroscopy. The ^{1}H NMR spectra (400 MHz) were acquired at different temperatures (±1 K) and fitted manually with simulated spectra of the DNMR6 algorithm as part of the iNMR software.[S5]

2. Product Characterization

Reaction of 4e with 2a. Dichloromethane solutions of **4e**-Cl (21.3 mg, 0.081 mmol, in 0.5 mL) and of dimethylsulfide (**2a**) (7.55 mg, 0.122 mmol, in 0.4 mL) were mixed. Then a solution of trimethylsilyl triflate (18.6 mg, 0.0834 mmol) in dichloromethane (0.5 mL) was added. Subsequently, volatiles were removed under reduced pressure. The viscous residue was analyzed by NMR spectroscopy, which showed quantitative conversion of **4e** into (**4e-SMe$_2$**)·TfO$^-$. **^{1}H NMR** (400 MHz, CD_2Cl_2): δ 7.56 (d, *J* = 8.8 Hz, 4 H, 3-H), 6.98 (d, *J* = 8.8 Hz, 4 H, 4-H), 6.11 (s, 1 H, 6-H), 3.81 (s, 6 H, 1-H), 2.77 (s, 6 H, 7-H). **^{13}C{^{1}H} NMR** (101 MHz, CD_2Cl_2): δ 161.6 (C_q, C-2), 130.6 (CH, C-3), 124.8 (C_q, C-5), 121.2 (C_q, q, *J* = 320 Hz, CF_3), 115.9 (CH, C-4), 66.5 (CH, C-6), 56.0 (CH_3, C-1), 24.3 (CH_3, C-7).

Reaction of 4e with 2b. Dichloromethane solutions of **4e**-Cl (21.3 mg, 0.081 mmol, in 0.5 mL) and of di-*n*-butylsulfide (**2b**) (11.9 mg, 0.081 mmol, in 0.25 mL) were mixed. Then a solution of trimethylsilyl triflate (18.6 mg, 0.0834 mmol) in dichloromethane (0.5 mL) was added. Subsequently, volatiles were removed under reduced pressure. The viscous residue was analyzed by NMR spectroscopy, which showed quantitative conversion of **4e** into (**4e-SBu$_2$**)·TfO$^-$. **^{1}H NMR** (400 MHz, CD_2Cl_2): δ 7.60 (d, *J* = 8.8 Hz, 4 H, 3-H), 6.99 (d, *J* = 8.8 Hz, 4 H, 4-H), 6.26 (s, 1 H, 6-H), 3.81 (s, 6 H, 1-H), 3.17 (br s, 4 H, 7-H), 1.47 (quint, *J* = 7.3 Hz, 4 H, 8-H), 1.37–1.28 (m, 4 H, 9-H), 0.83 (t, *J* = 7.3 Hz, 6 H, 10-H). **^{13}C{^{1}H} NMR** (101

MHz, CD_2Cl_2): δ 161.6 (C_q, C-2), 130.7 (CH, C-3), 125.7 (C_q, C-5), 121.2 (C_q, q, J = 320 Hz, CF_3), 115.8 (CH, C-4), 66.0 (CH, C-6), 56.0 (CH_3, C-1), 40.3 (CH_2, C-7), 28.4 (CH_2, C-8), 22.1 (CH_2, C-9), 13.5 (CH_3, C-10).

Reaction of 4e with 2c. Dichloromethane solutions of **4e**-Cl (21.3 mg, 0.081 mmol, in 0.5 mL) and of THT (**2c**) (7.14 mg, 0.081 mmol, in 0.25 mL) were mixed. Then a solution of trimethylsilyl triflate (18.6 mg, 0.0834 mmol) in dichloromethane (0.5 mL) was added. Subsequently, volatiles were removed under reduced pressure. The viscous residue was analyzed by NMR spectroscopy, which showed quantitative conversion of **4e** into (**4e-THT**)·TfO^-. **^{1}H NMR** (400 MHz, CD_2Cl_2): δ 7.56 (d, J = 8.8 Hz, 4 H, 3-H), 6.97 (d, J = 8.8 Hz, 4 H, 4-H), 5.78 (s, 1 H, 6-H), 3.80 (s, 6 H, 1-H), 3.28 (br s, 4 H, 7-H), 2.41 (br s, 4 H, 8-H). **^{13}C{^{1}H} NMR** (101 MHz, CD_2Cl_2): δ 161.6 (C_q, C-2), 130.7 (CH, C-3), 125.9 (C_q, C-5), 121.4 (C_q, q, J = 321 Hz, CF_3), 115.9 (CH, C-4), 65.2 (CH, C-6), 56.0 (CH_3, C-1), 42.9 (CH_2, C-7), 29.6 (CH_2, C-8).

Reaction of 4e with 2d. Dichloromethane solutions of **4e**-Cl (21.3 mg, 0.081 mmol, in 0.5 mL) and of THTP (**2d**) (8.28 mg, 0.081 mmol, in 0.25 mL) were mixed. Then a solution of trimethylsilyl triflate (18.6 mg, 0.0834 mmol) in dichloromethane (0.5 mL) was added. Subsequently, volatiles were removed under reduced pressure. The viscous residue was analyzed by NMR spectroscopy, which showed quantitative conversion of **4e** into (**4e-THTP**)·TfO^-. **^{1}H NMR** (400 MHz, CD_2Cl_2): δ 7.57 (d, J = 8.8 Hz, 4 H,3-H), 6.98 (d, J = 8.8 Hz, 4 H, 4-H), 6.28 (s, 1 H, 6-H), 3.80 (s, 6 H, 1-H), 3.22 (s, 4 H, 7-H), 1.98 (s, 4 H, 8-H), 1.82–1.75 (m, 2 H, 9-H). **^{13}C{^{1}H} NMR** (101 MHz, CD_2Cl_2): δ 161.5 (C_q, C-2), 130.5 (CH, C-3), 124.7 (C_q, C-5), 121.4 (C_q, q, J = 320 Hz, CF_3), 115.8 (CH, C-4), 64.8 (CH, C-6), 56.0 (CH_3, C-1), 37.2 (CH_2, C-7), 23.8 (CH_2, C-8), 23.1 (CH_2, C-9).

3. Kinetics of the reactions of chalcogenides with benzhydrylium ions

3.1 Kinetics of the reactions of Me_2S (2a) with the Ar_2CH^+ (4) in CH_2Cl_2

Kinetics of the reaction of **2a** with (fur)$_2$CH$^+$ **4g** at 20 °C in CH_2Cl_2 (Laser-flash photolysis, λ = 534 nm)

[**4g**-PPh$_3$BF$_4$] (mol L^{-1})	[**2a**] (mol L^{-1})	k_{obs} (s^{-1})
3.00 × 10^{-5}	7.24 × 10^{-4}	1.81 × 10^{5}
3.00 × 10^{-5}	1.09 × 10^{-3}	2.13 × 10^{5}
3.00 × 10^{-5}	1.45 × 10^{-3}	2.45 × 10^{5}
3.00 × 10^{-5}	1.81 × 10^{-3}	2.75 × 10^{5}
3.00 × 10^{-5}	2.17 × 10^{-3}	2.97 × 10^{5}

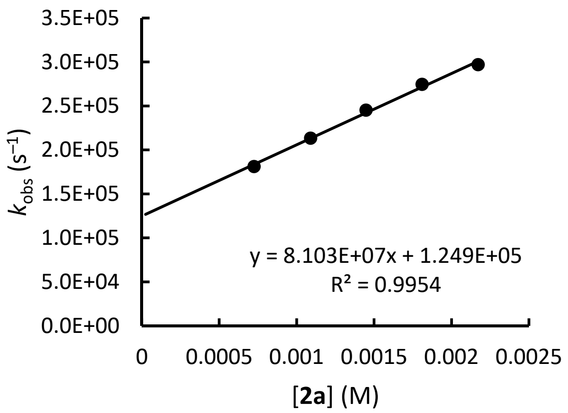

k = 8.10 × 10^{7} M^{-1} s^{-1}

Kinetics of the reaction of **2a** with (fur)(ani)CH$^+$ **4f** at 20 °C in CH_2Cl_2 (Laser-flash photolysis, λ = 524 nm)

[**4f**-PPh$_3$BF$_4$ (mol L^{-1})	[**2a**] (mol L^{-1})	k_{obs} (s^{-1})
2.86 × 10^{-5}	7.21 × 10^{-4}	2.19 × 10^{5}
2.86 × 10^{-5}	1.08 × 10^{-3}	3.00 × 10^{5}
2.86 × 10^{-5}	1.44 × 10^{-3}	3.65 × 10^{5}
2.86 × 10^{-5}	1.80 × 10^{-3}	4.42 × 10^{5}
2.86 × 10^{-5}	2.16 × 10^{-3}	5.12 × 10^{5}

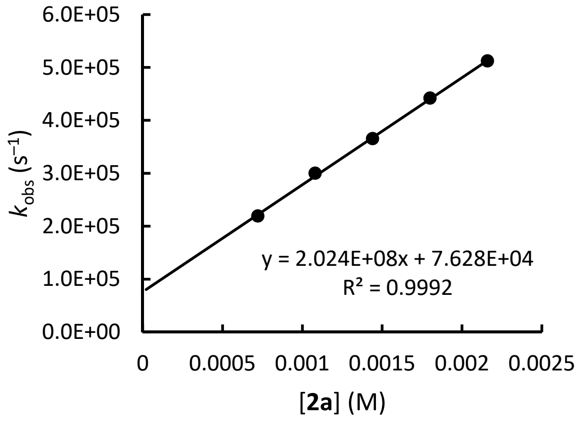

k = 2.02 × 10^{8} M^{-1} s^{-1}

Kinetics of the reaction of **2a** with (ani)$_2$CH$^+$ **4e** at 20 °C in CH_2Cl_2 (Laser-flash photolysis, λ = 513 nm)

[**4e**-PPh$_3$BF$_4$] (mol L^{-1})	[**2a**] (mol L^{-1})	k_{obs} (s^{-1})
3.12 × 10^{-5}	7.24 × 10^{-4}	3.93 × 10^{5}
3.12 × 10^{-5}	1.09 × 10^{-3}	5.89 × 10^{5}
3.12 × 10^{-5}	1.45 × 10^{-3}	7.46 × 10^{5}
3.12 × 10^{-5}	1.81 × 10^{-3}	9.34 × 10^{5}
3.12 × 10^{-5}	2.17 × 10^{-3}	1.15 × 10^{6}

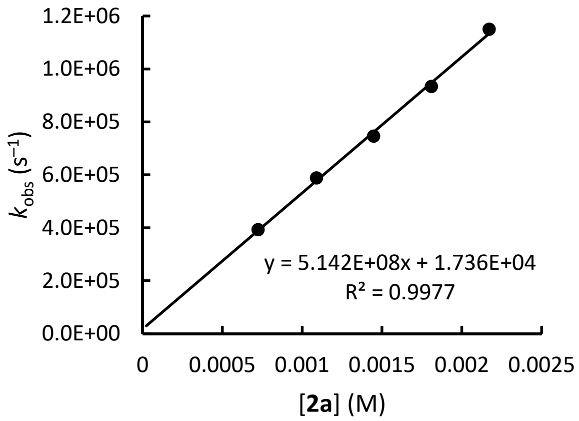

k = 5.14 × 10^{8} M^{-1} s^{-1}

Kinetics of the reaction of **2a** with (ani)(pop)CH$^+$ **4d** at 20 °C in CH_2Cl_2 (Laser-flash photolysis, λ = 512 nm)

[**4d**-PPh_3BF_4] (mol L^{-1})	[**2a**] (mol L^{-1})	k_{obs} (s^{-1})
3.20×10^{-5}	7.21×10^{-4}	6.44×10^{5}
3.20×10^{-5}	1.08×10^{-3}	9.56×10^{5}
3.20×10^{-5}	1.44×10^{-3}	1.25×10^{6}
3.20×10^{-5}	1.80×10^{-3}	1.58×10^{6}
3.20×10^{-5}	2.16×10^{-3}	1.92×10^{5}

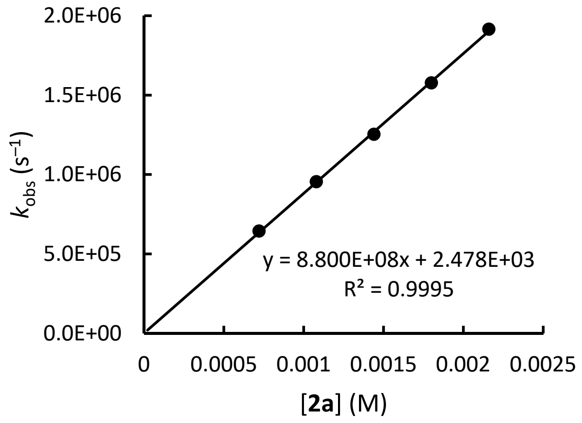

***k* = 8.80 × 10^{8} M^{-1} s^{-1}**

3.2 Kinetics of the reactions of Me_2S (2a) with the Ar_2CH^+ (4) in MeCN

Kinetics of the reaction of **2a** with $(fur)_2CH^+$ **4g** at 20 °C in MeCN (Laser-flash photolysis, λ = 523 nm)

[**4g**-PPh_3BF_4] (mol L^{-1})	[**2a**] (mol L^{-1})	k_{obs} (s^{-1})
3.80×10^{-5}	1.78×10^{-4}	4.81×10^{4}
3.80×10^{-5}	2.67×10^{-4}	6.30×10^{4}
3.80×10^{-5}	3.56×10^{-4}	7.56×10^{4}
3.80×10^{-5}	4.45×10^{-4}	8.91×10^{4}
3.80×10^{-5}	5.34×10^{-4}	1.04×10^{5}

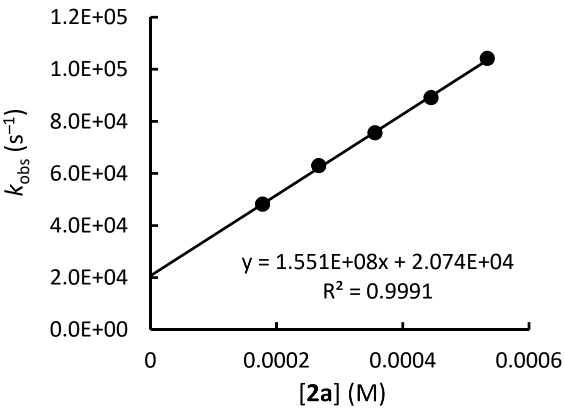

***k* = 1.55 × 10^{8} M^{-1} s^{-1}**

Kinetics of the reaction of **2a** with (fur)(ani)CH$^+$ **4f** at 20 °C in MeCN (Laser-flash photolysis, λ = 513 nm)

[**4f**-PPh_3BF_4] (mol L^{-1})	[**2a**] (mol L^{-1})	k_{obs} (s^{-1})
2.72×10^{-5}	1.50×10^{-4}	6.85×10^{5}
2.72×10^{-5}	2.25×10^{-4}	9.76×10^{5}
2.72×10^{-5}	3.00×10^{-4}	1.23×10^{5}
2.72×10^{-5}	3.75×10^{-4}	1.46×10^{5}
2.72×10^{-5}	4.50×10^{-4}	1.68×10^{5}

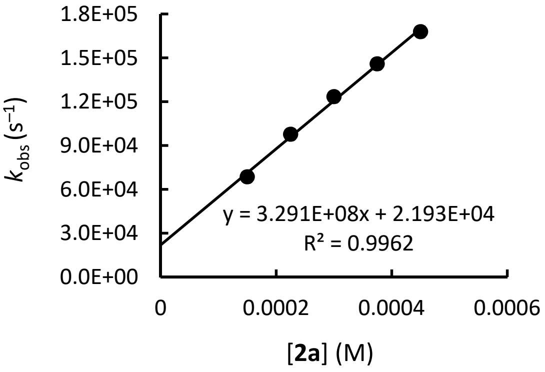

***k* = 3.29 × 10^{8} M^{-1} s^{-1}**

Kinetics of the reaction of **2a** with $(ani)_2CH^+$ **4e** at 20 °C in MeCN (Laser-flash photolysis, λ = 500 nm)

[**4e**-PPh_3BF_4] (mol L^{-1})	[**2a**] (mol L^{-1})	k_{obs} (s^{-1})
3.12×10^{-5}	1.78×10^{-4}	1.58×10^{5}
3.12×10^{-5}	2.67×10^{-4}	2.28×10^{5}
3.12×10^{-5}	3.56×10^{-4}	2.90×10^{5}
3.12×10^{-5}	4.45×10^{-4}	3.56×10^{5}
3.12×10^{-5}	5.34×10^{-4}	4.26×10^{5}

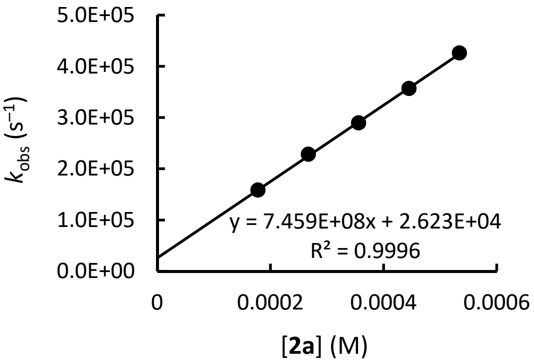

$\boldsymbol{k = 7.46 \times 10^{8}\ M^{-1}\ s^{-1}}$

Kinetics of the reaction of **2a** with (ani)(pop)CH^+ **4d** at 20 °C in MeCN (Laser-flash photolysis, λ = 500 nm)

[**4d**-PPh_3BF_4] (mol L^{-1})	[**2a**] (mol L^{-1})	k_{obs} (s^{-1})
3.20×10^{-5}	2.29×10^{-4}	3.35×10^{5}
3.20×10^{-5}	3.44×10^{-4}	4.84×10^{5}
3.20×10^{-5}	4.58×10^{-4}	6.10×10^{5}
3.20×10^{-5}	5.73×10^{-4}	7.56×10^{5}
3.20×10^{-5}	6.87×10^{-4}	8.98×10^{5}

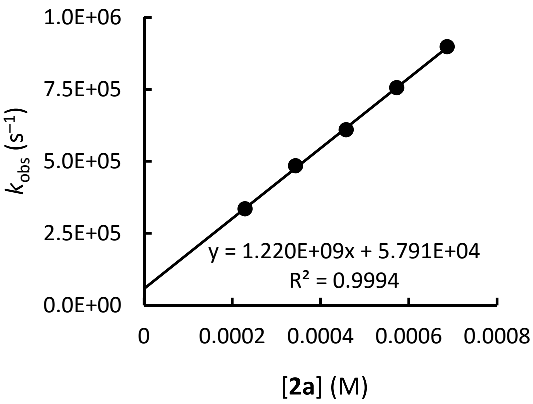

$\boldsymbol{k = 1.22 \times 10^{9}\ M^{-1}\ s^{-1}}$

Kinetics of the reaction of **2a** with (ani)(Ph)CH^+ **4b** at 20 °C in MeCN (Laser-flash photolysis, λ = 455 nm)

[**4b**-PPh_3BF_4] (mol L^{-1})	[**2a**] (mol L^{-1})	k_{obs} (s^{-1})
3.20×10^{-5}	2.88×10^{-4}	9.52×10^{5}
3.20×10^{-5}	4.32×10^{-4}	1.33×10^{6}
3.20×10^{-5}	5.76×10^{-4}	1.81×10^{6}
3.20×10^{-5}	7.20×10^{-4}	2.22×10^{6}
3.20×10^{-5}	8.64×10^{-4}	2.57×10^{6}

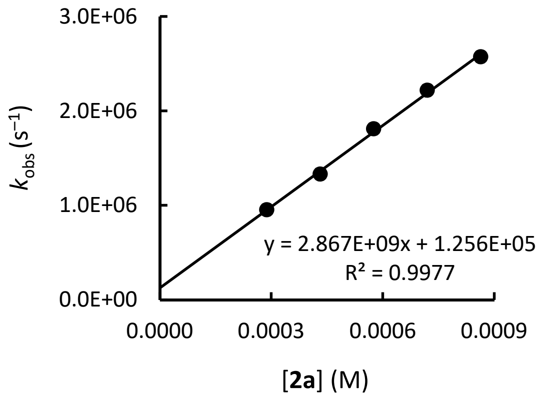

$\boldsymbol{k = 2.87 \times 10^{9}\ M^{-1}\ s^{-1}}$

3.3 Kinetics of the reactions of *n*Bu_2S (2b) with the Ar_2CH^+ (4) in CH_2Cl_2

Kinetics of the reaction of **2b** with (fur)$_2CH^+$ **4g** at 20 °C in CH_2Cl_2 (Laser-flash photolysis, λ = 530 nm)

[**4g**-PPh_3BF_4] (mol L^{-1})	[**2b**] (mol L^{-1})	k_{obs} (s^{-1})
3.72×10^{-5}	1.29×10^{-3}	2.22×10^{5}
3.72×10^{-5}	2.57×10^{-3}	2.82×10^{5}
3.72×10^{-5}	3.22×10^{-3}	3.09×10^{5}
3.72×10^{-5}	3.86×10^{-3}	3.60×10^{5}
3.72×10^{-5}	5.15×10^{-3}	4.16×10^{5}

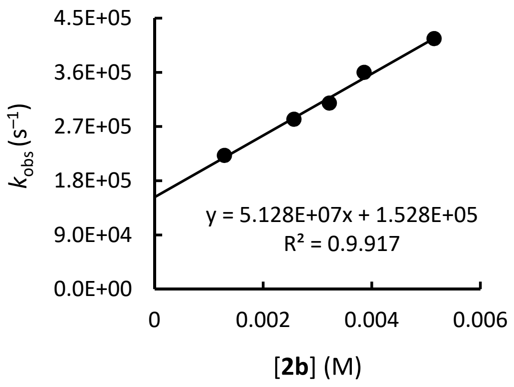

$k = 5.13 \times 10^{7}$ M^{-1} s^{-1}

Kinetics of the reaction of **2b** with (fur)(ani)CH^+ **4f** at 20 °C in CH_2Cl_2 (Laser-flash photolysis, λ = 528 nm)

[**4f**-PPh_3BF_4] (mol L^{-1})	[**2b**] (mol L^{-1})	k_{obs} (s^{-1})
2.26×10^{-5}	1.12×10^{-3}	2.76×10^{5}
2.26×10^{-5}	2.24×10^{-3}	4.40×10^{5}
2.26×10^{-5}	3.36×10^{-3}	5.94×10^{5}
2.26×10^{-5}	4.48×10^{-3}	7.33×10^{5}
2.26×10^{-5}	5.59×10^{-3}	8.54×10^{5}

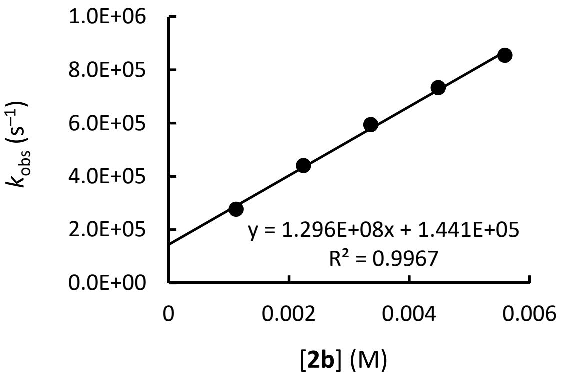

$k = 1.30 \times 10^{8}$ M^{-1} s^{-1}

Kinetics of the reaction of **2b** with (ani)$_2CH^+$ **4e** at 20 °C in CH_2Cl_2 (Laser-flash photolysis, λ = 515 nm)

[**4e**-PPh_3BF_4] (mol L^{-1})	[**2b**] (mol L^{-1})	k_{obs} (s^{-1})
1.29×10^{-5}	1.29×10^{-3}	3.79×10^{5}
1.29×10^{-5}	2.57×10^{-3}	7.46×10^{5}
1.29×10^{-5}	3.22×10^{-3}	8.99×10^{5}
1.29×10^{-5}	3.86×10^{-3}	1.07×10^{6}
1.29×10^{-5}	5.15×10^{-3}	1.44×10^{6}

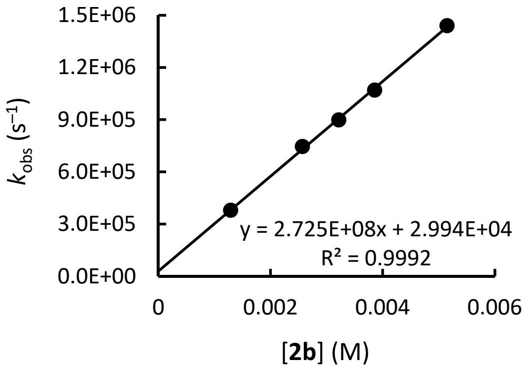

$k = 2.73 \times 10^{8}$ M^{-1} s^{-1}

Kinetics of the reaction of **2b** with (ani)(pop)CH^+ **4d** at 20 °C in CH_2Cl_2 (Laser-flash photolysis, λ = 516 nm)

[**4d**-PPh_3BF_4] (mol L^{-1})	[**2b**] (mol L^{-1})	k_{obs} (s^{-1})
1.50×10^{-5}	5.59×10^{-4}	2.69×10^{5}
1.50×10^{-5}	1.12×10^{-3}	5.30×10^{5}
1.50×10^{-5}	1.68×10^{-3}	7.74×10^{5}
1.50×10^{-5}	2.24×10^{-3}	1.05×10^{6}
1.50×10^{-5}	2.80×10^{-3}	1.30×10^{6}

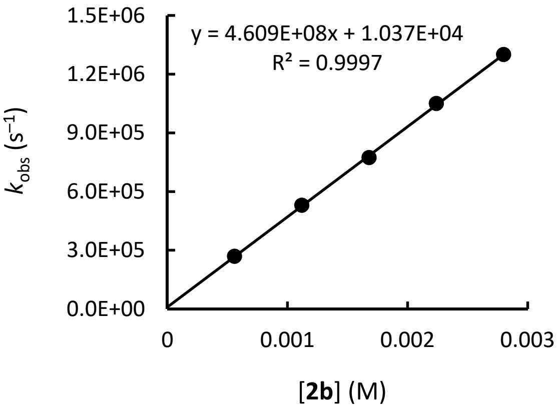

***k* = 4.61 × 10^8 M^{-1} s^{-1}**

3.4 Kinetics of the reactions of tetrahydrothiophene (2c) with the Ar_2CH^+ (4) in CH_2Cl_2

Kinetics of the reaction of **2c** with $(fur)_2CH^+$ **4g** at 20 °C in CH_2Cl_2 (Laser-flash photolysis, λ = 534 nm)

[**4g**-PPh_3BF_4] (mol L^{-1})	[**2c**] (mol L^{-1})	k_{obs} (s^{-1})
3.00×10^{-5}	6.65×10^{-4}	5.06×10^{5}
3.00×10^{-5}	9.98×10^{-4}	6.32×10^{5}
3.00×10^{-5}	1.33×10^{-3}	7.14×10^{5}
3.00×10^{-5}	1.66×10^{-3}	7.81×10^{5}
3.00×10^{-5}	2.00×10^{-3}	9.10×10^{5}

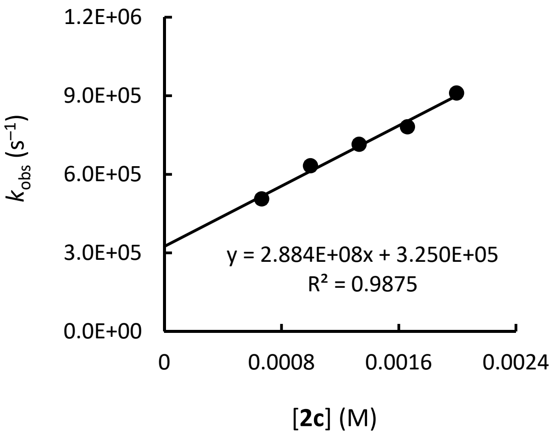

***k* = 2.88 × 10^8 M^{-1} s^{-1}**

Kinetics of the reaction of **2c** with (fur)(ani)CH^+ **4f** at 20 °C in CH_2Cl_2 (Laser-flash photolysis, λ = 524 nm)

[**4f**-PPh_3BF_4] (mol L^{-1})	[**2c**] (mol L^{-1})	k_{obs} (s^{-1})
2.86×10^{-5}	6.67×10^{-4}	4.35×10^{5}
2.86×10^{-5}	1.00×10^{-3}	5.75×10^{5}
2.86×10^{-5}	1.33×10^{-3}	6.89×10^{5}
2.86×10^{-5}	1.67×10^{-3}	8.25×10^{5}
2.86×10^{-5}	2.00×10^{-3}	9.52×10^{5}

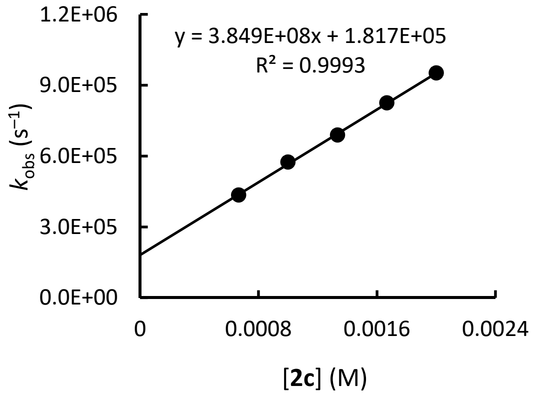

***k* = 3.85 × 10^8^ M^–1^ s^–1^**

Kinetics of the reaction of **2c** with $(ani)_2CH^+$ **4e** at 20 °C in CH_2Cl_2 (Laser-flash photolysis, λ = 513 nm)

[**4e**-PPh_3BF_4] (mol L^{-1})	[**2c**] (mol L^{-1})	k_{obs} (s^{-1})
3.12×10^{-5}	6.65×10^{-4}	5.85×10^{5}
3.12×10^{-5}	9.98×10^{-4}	8.72×10^{5}
3.12×10^{-5}	1.33×10^{-3}	1.13×10^{6}
3.12×10^{-5}	1.66×10^{-3}	1.41×10^{6}
3.12×10^{-5}	2.00×10^{-3}	1.67×10^{5}

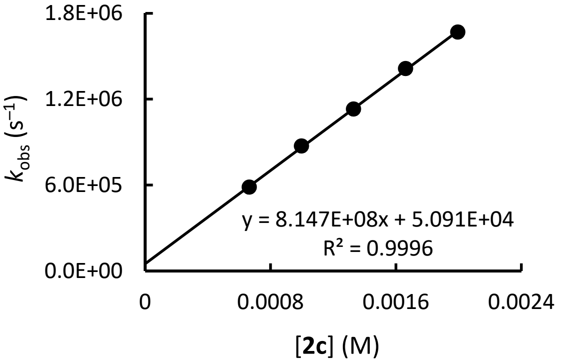

k = 8.15 × 10^8 M^{-1} s^{-1}

Kinetics of the reaction of **2c** with (ani)(pop)CH^+ **4d** at 20 °C in CH_2Cl_2 (Laser-flash photolysis, λ = 512 nm)

[**4d**-PPh_3BF_4] (mol L^{-1})	[**2c**] (mol L^{-1})	k_{obs} (s^{-1})
3.20×10^{-5}	6.67×10^{-4}	8.43×10^{5}
3.20×10^{-5}	1.00×10^{-3}	1.29×10^{6}
3.20×10^{-5}	1.33×10^{-3}	1.64×10^{6}
3.20×10^{-5}	1.67×10^{-3}	2.11×10^{6}
3.20×10^{-5}	2.00×10^{-3}	2.47×10^{6}

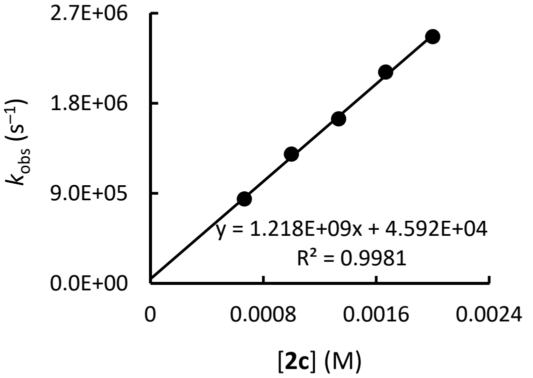

k = 1.22 × 10^9 M^{-1} s^{-1}

3.5 Kinetics of the reactions of tetrahydrothiophene (2c) with the Ar_2CH^+ (4) in MeCN

Kinetics of the reaction of **2c** with $(fur)_2CH^+$ **4g** at 20 °C in CH_3CN (Laser-flash photolysis, λ = 523 nm)

[**4g**-PPh_3BF_4] (mol L^{-1})	[**2c**] (mol L^{-1})	k_{obs} (s^{-1})
3.80×10^{-5}	1.72×10^{-4}	9.60×10^{4}
3.80×10^{-5}	2.58×10^{-4}	1.31×10^{5}
3.80×10^{-5}	3.44×10^{-4}	1.72×10^{5}
3.80×10^{-5}	4.30×10^{-4}	2.02×10^{5}
3.80×10^{-5}	5.16×10^{-4}	2.32×10^{5}

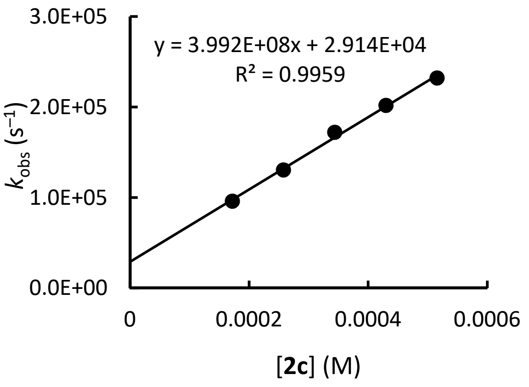

***k* = 3.99 × 10^8^ M^–1^ s^–1^**

Kinetics of the reaction of **2c** with (fur)(ani)CH^+ **4f** at 20 °C in MeCN (Laser-flash photolysis, λ = 513 nm)

[**4f**-PPh_3BF_4] (mol L^{-1})	[**2c**] (mol L^{-1})	k_{obs} (s^{-1})
2.72×10^{-5}	1.50×10^{-4}	1.38×10^{5}
2.72×10^{-5}	2.25×10^{-4}	1.92×10^{5}
2.72×10^{-5}	3.00×10^{-4}	2.40×10^{5}
2.72×10^{-5}	3.75×10^{-4}	2.95×10^{5}
2.72×10^{-5}	4.50×10^{-4}	3.45×10^{5}

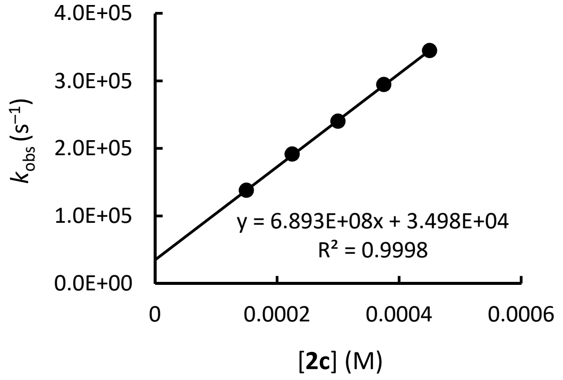

k = 6.89 × 10^8 M^{-1} s^{-1}

Kinetics of the reaction of **2c** with $(ani)_2CH^+$ **4e** at 20 °C in MeCN (Laser-flash photolysis, λ = 500 nm)

[**4e**-PPh_3BF_4] (mol L^{-1})	[**2c**] (mol L^{-1})	k_{obs} (s^{-1})
3.12×10^{-5}	1.72×10^{-4}	3.25×10^{5}
3.12×10^{-5}	2.58×10^{-4}	4.62×10^{5}
3.12×10^{-5}	3.44×10^{-4}	6.16×10^{5}
3.12×10^{-5}	4.30×10^{-4}	7.70×10^{5}
3.12×10^{-5}	5.16×10^{-4}	8.81×10^{5}

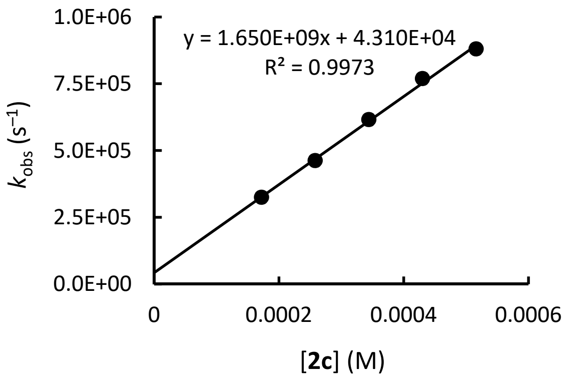

k = 1.65 × 10^9 M^{-1} s^{-1}

Kinetics of the reaction of **2c** with (ani)(pop)CH$^+$ **4d** at 20 °C in MeCN (Laser-flash photolysis, λ = 500 nm)

[**4d**-PPh_3BF_4] (mol L^{-1})	[**2c**] (mol L^{-1})	k_{obs} (s^{-1})
3.20×10^{-5}	1.88×10^{-4}	3.67×10^{5}
3.20×10^{-5}	2.82×10^{-4}	5.37×10^{5}
3.20×10^{-5}	3.76×10^{-4}	6.90×10^{5}
3.20×10^{-5}	4.70×10^{-4}	8.50×10^{5}
3.20×10^{-5}	5.64×10^{-4}	1.00×10^{6}

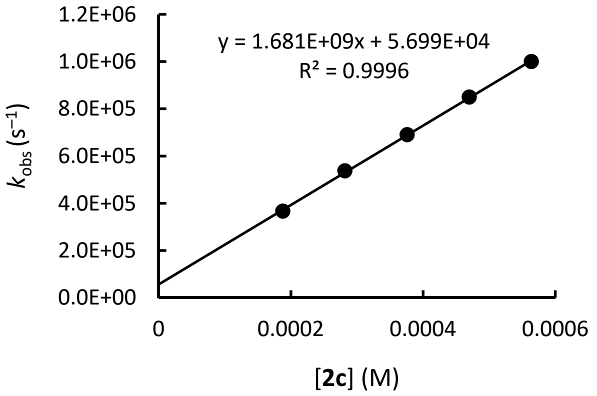

$\boldsymbol{k = 1.68 \times 10^9\ M^{-1}\ s^{-1}}$

Kinetics of the reaction of **2c** with (ani)(Ph)CH$^+$ **4b** at 20 °C in MeCN (Laser-flash photolysis, λ = 455 nm)

[**4b**-PPh_3BF_4] (mol L^{-1})	[**2c**] (mol L^{-1})	k_{obs} (s^{-1})
5.71×10^{-5}	2.99×10^{-4}	1.59×10^{6}
5.71×10^{-5}	4.49×10^{-4}	2.41×10^{6}
5.71×10^{-5}	5.98×10^{-4}	3.13×10^{6}
5.71×10^{-5}	7.48×10^{-4}	3.83×10^{6}
5.71×10^{-5}	8.97×10^{-4}	4.58×10^{6}

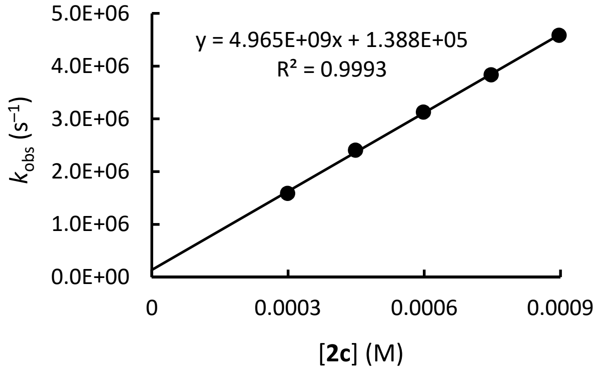

$\boldsymbol{k = 4.97 \times 10^9\ M^{-1}\ s^{-1}}$

Kinetics of the reaction of **2c** with (pop)(Ph)CH$^+$ **4a** at 20 °C in MeCN (Laser-flash photolysis, λ = 466 nm)

[**4a**-PPh_3BF_4] (mol L^{-1})	[**2c**] (mol L^{-1})	k_{obs} (s^{-1})
5.13×10^{-5}	3.08×10^{-4}	1.82×10^{6}
5.12×10^{-5}	4.62×10^{-4}	2.80×10^{6}
5.13×10^{-5}	6.16×10^{-4}	3.58×10^{6}
5.13×10^{-5}	7.70×10^{-4}	4.38×10^{6}
5.13×10^{-5}	9.24×10^{-4}	5.01×10^{6}

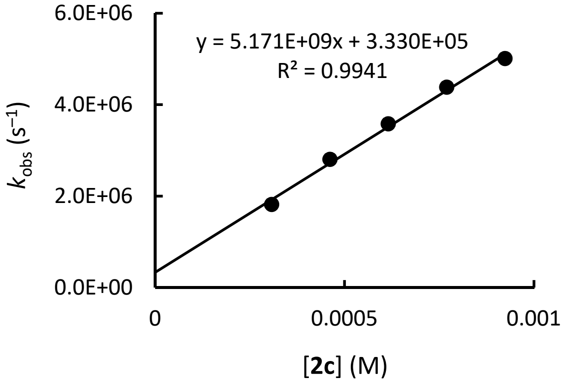

$\boldsymbol{k = 5.17 \times 10^9\ M^{-1}\ s^{-1}}$

3.6 Kinetics of the reactions of tetrahydro-2*H*-thiopyran (2d) with the Ar_2CH^+ (4) in CH_2Cl_2

Kinetics of the reaction of **2d** with $(fur)_2CH^+$ **4g** at 20 °C in CH_2Cl_2 (Laser-flash photolysis, λ = 530 nm)

[**4g**-PPh_3BF_4] (mol L^{-1})	[**2d**] (mol L^{-1})	k_{obs} (s^{-1})
1.86×10^{-5}	1.44×10^{-3}	2.25×10^{5}
1.86×10^{-5}	2.16×10^{-3}	2.72×10^{5}
1.86×10^{-5}	2.88×10^{-3}	3.49×10^{5}
1.86×10^{-5}	4.32×10^{-3}	4.52×10^{5}
1.86×10^{-5}	5.75×10^{-3}	5.86×10^{5}

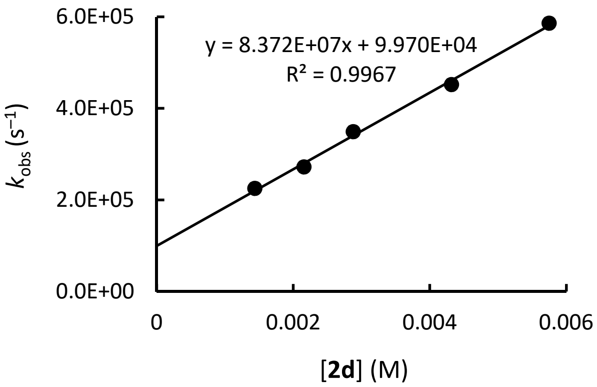

***k* = 8.37 × 10^7^ M^–1^ s^–1^**

$k = 8.37 \times 10^{7}$ M^{-1} s^{-1}

Kinetics of the reaction of **2d** with $(fur)(ani)CH^+$ **4f** at 20 °C in CH_2Cl_2 (Laser-flash photolysis, λ = 528 nm)

[**4f**-PPh_3BF_4] (mol L^{-1})	[**2d**] (mol L^{-1})	k_{obs} (s^{-1})
1.29×10^{-5}	7.19×10^{-4}	2.19×10^{5}
1.29×10^{-5}	1.44×10^{-3}	3.71×10^{5}
1.29×10^{-5}	2.16×10^{-3}	5.31×10^{5}
1.29×10^{-5}	2.88×10^{-3}	6.84×10^{5}
1.29×10^{-5}	3.60×10^{-3}	8.41×10^{5}

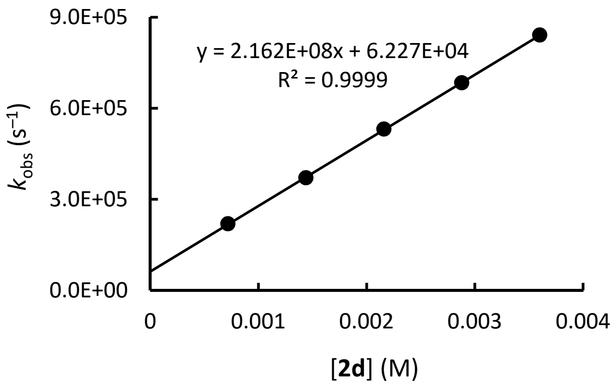

$k = 2.16 \times 10^{8}$ M^{-1} s^{-1}

Kinetics of the reaction of **2d** with $(ani)_2CH^+$ **4e** at 20 °C in CH_2Cl_2 (Laser-flash photolysis, λ = 515 nm)

[**4e**-PPh_3BF_4] (mol L^{-1})	[**2d**] (mol L^{-1})	k_{obs} (s^{-1})
2.26×10^{-5}	5.13×10^{-4}	2.81×10^{5}
2.26×10^{-5}	1.03×10^{-3}	5.79×10^{5}
2.26×10^{-5}	1.54×10^{-3}	8.69×10^{5}
2.26×10^{-5}	2.05×10^{-3}	1.13×10^{6}
2.26×10^{-5}	2.56×10^{-3}	1.43×10^{6}

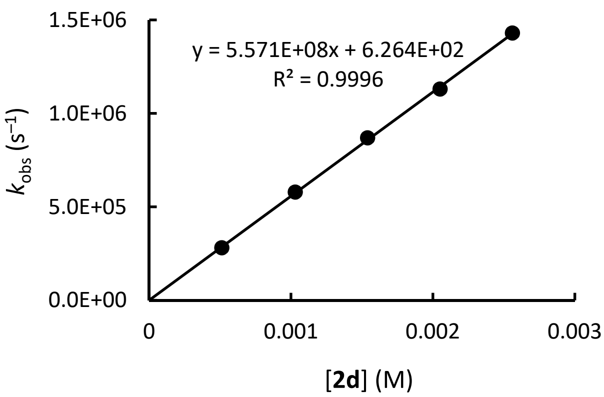

$k = 5.57 \times 10^{8}$ M^{-1} s^{-1}

Kinetics of the reaction of **2d** with (ani)(pop)CH$^+$ **4d** at 20 °C in CH_2Cl_2 (Laser-flash photolysis, λ = 515 nm)

[**4d**-PPh_3BF_4] (mol L^{-1})	[**2d**] (mol L^{-1})	k_{obs} (s^{-1})
1.50×10^{-5}	5.13×10^{-4}	4.19×10^{5}
1.50×10^{-5}	1.03×10^{-3}	8.27×10^{5}
1.50×10^{-5}	1.54×10^{-3}	1.21×10^{6}
1.50×10^{-5}	2.05×10^{-3}	1.58×10^{6}
1.50×10^{-5}	2.56×10^{-3}	1.92×10^{6}

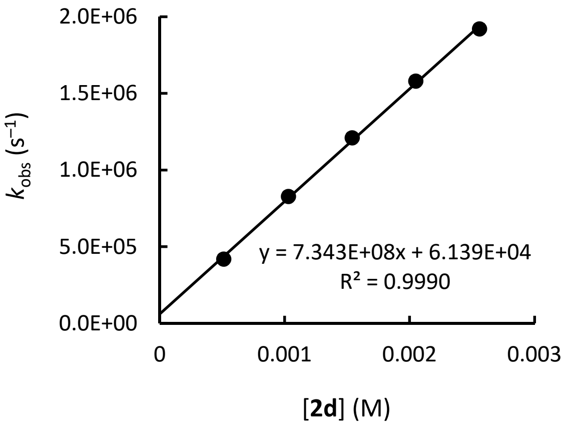

***k* = 7.34 × 10^8 M^{-1} s^{-1}**

3.7 Kinetics of the reactions of Me_2Se (3) with the Ar_2CH^+ (4) in CH_2Cl_2

Kinetics of the reaction of **3** with (fur)(ani)CH$^+$ **4f** at 20 °C in CH_2Cl_2 (Laser-flash photolysis, λ = 528 nm)

[**4f**-PPh_3BF_4 (mol L^{-1})	[**3**] (mol L^{-1})	k_{obs} (s^{-1})
1.28×10^{-5}	9.61×10^{-4}	5.94×10^{5}
1.28×10^{-5}	1.44×10^{-3}	7.47×10^{5}
1.28×10^{-5}	1.92×10^{-3}	9.73×10^{5}
1.28×10^{-5}	2.40×10^{-3}	1.05×10^{6}
1.28×10^{-5}	2.88×10^{-3}	1.20×10^{6}

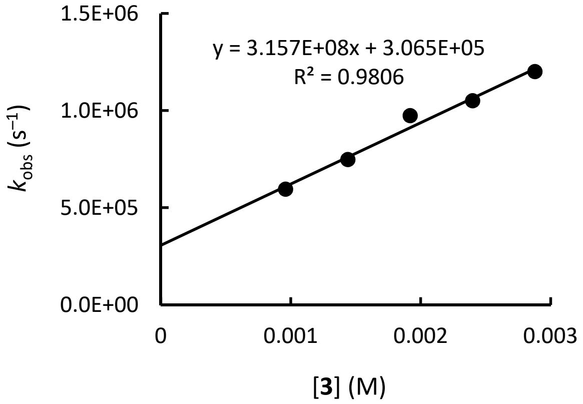

***k* = 3.16 × 10^8 M^{-1} s^{-1}**

Kinetics of the reaction of **3** with $(ani)_2CH^+$ **4e** at 20 °C in CH_2Cl_2 (Laser-flash photolysis, λ = 513 nm)

[**4e**-PPh_3BF_4] (mol L^{-1})	[**3**] (mol L^{-1})	k_{obs} (s^{-1})
1.39×10^{-5}	9.61×10^{-4}	5.94×10^{5}
1.39×10^{-5}	1.44×10^{-3}	7.47×10^{5}
1.39×10^{-5}	1.92×10^{-3}	9.73×10^{5}
1.39×10^{-5}	2.40×10^{-3}	1.05×10^{6}
1.39×10^{-5}	2.88×10^{-3}	1.20×10^{6}

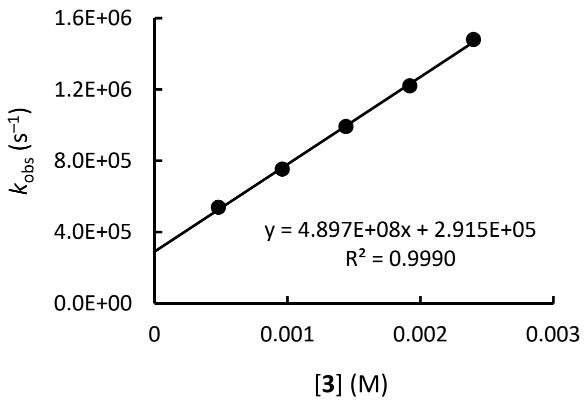

***k* = 4.90 × 10^{8} M^{-1} s^{-1}**

Kinetics of the reaction of **3** with (ani)(pop)CH^+ **4d** at 20 °C in CH_2Cl_2 (Laser-flash photolysis, λ = 516 nm)

[**4d**-PPh_3BF_4] (mol L^{-1})	[**3**] (mol L^{-1})	k_{obs} (s^{-1})
1.38×10^{-5}	6.31×10^{-4}	6.40×10^{5}
1.38×10^{-5}	9.46×10^{-4}	9.42×10^{5}
1.38×10^{-5}	1.26×10^{-3}	1.26×10^{6}
1.38×10^{-5}	1.58×10^{-3}	1.63×10^{6}
1.38×10^{-5}	1.89×10^{-3}	2.00×10^{6}

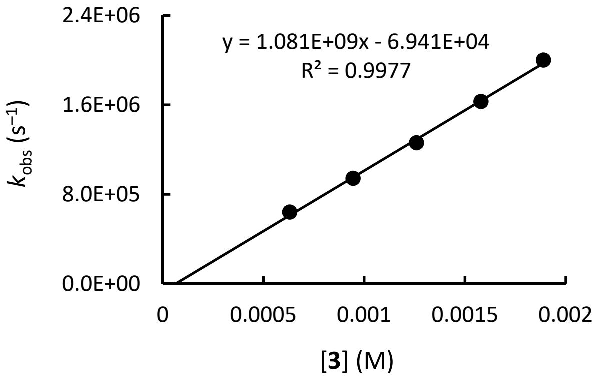

***k* = 1.08 × 10^{9} M^{-1} s^{-1}**

Kinetics of the reaction of **3** with (ani)(tol)CH^+ **4c** at 20 °C in CH_2Cl_2 (Laser-flash photolysis, λ = 516 nm)

[**4c**-PPh_3BF_4] (mol L^{-1})	[**3**] (mol L^{-1})	k_{obs} (s^{-1})
1.39×10^{-5}	3.16×10^{-4}	6.05×10^{5}
1.39×10^{-5}	6.30×10^{-4}	1.41×10^{6}
1.39×10^{-5}	8.76×10^{-4}	1.86×10^{6}
1.39×10^{-5}	1.26×10^{-3}	2.57×10^{6}
1.39×10^{-5}	1.58×10^{-3}	3.28×10^{6}

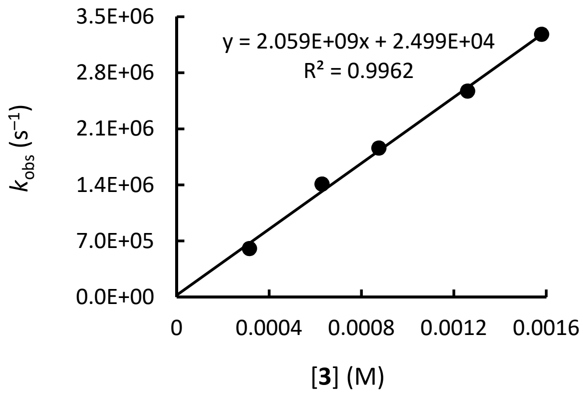

***k* = 2.06 × 10^{9} M^{-1} s^{-1}**

4. Determination of nucleofugality parameters N_f and s_f for **2** and **3**

Me_2S (**2a**)
$N_f = 6.33$; $s_f = 0.75$

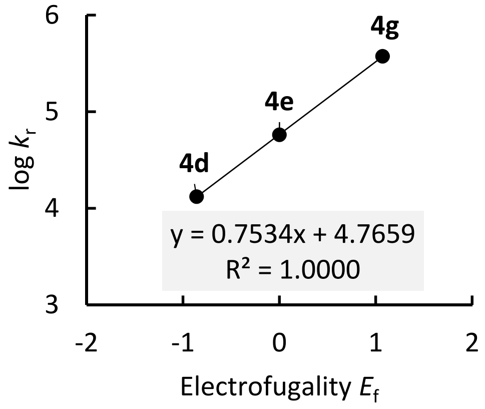

nBu_2S (**2b**)
$N_f = 6.36$; $s_f = 0.74$

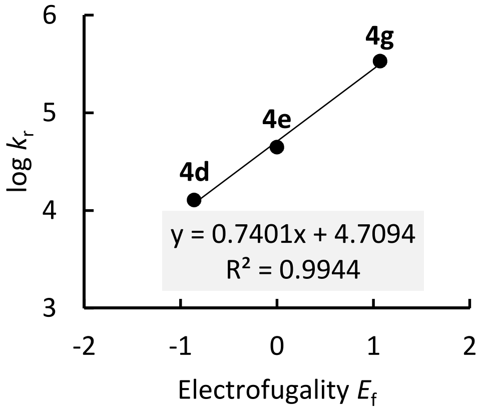

Tetrahydrothiophen (**2c**)
$N_f = 7.26$; $s_f = 0.66$

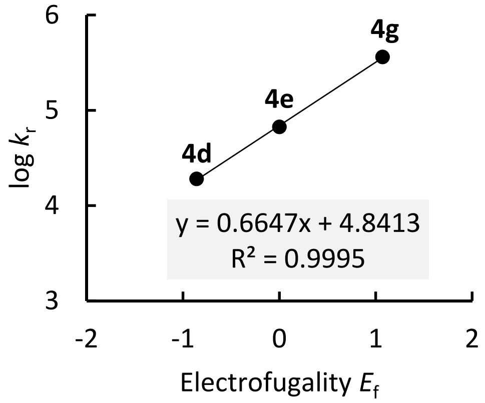

THTP (**2d**)
$N_f = 7.33$; $s_f = 0.59$

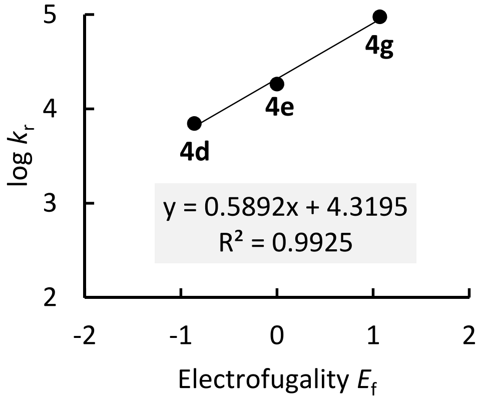

Me_2Se (**3**)
$N_f = 8.72$; $s_f = 0.66$

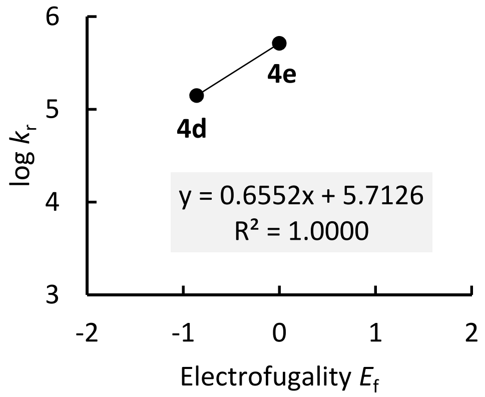

5. Dynamic ^{1}H NMR Spectroscopy and Line Shape Analysis

3.1 Dynamics of the Me_2S exchange in a CD_2Cl_2 mixture of (bis(2,3-dihydrobenzofuran-5-yl)methyl)-dimethylsulfonium triflate (4g-SMe_2 TfO^-) and dimethyl sulfide (2a)
(PJ147)

Generation of (**4g-SMe_2**) triflate in CD_2Cl_2 solution: **4g-Cl** (0.107 M, 1 equiv.), dimethylsulfide (**2a**, 0.16 M, 1.5 equiv.), and trimethylsilyl triflate (TMSOTf, 0.111 M, 1.05 equiv.) were dissolved in 0.7 mL CD_2Cl_2.

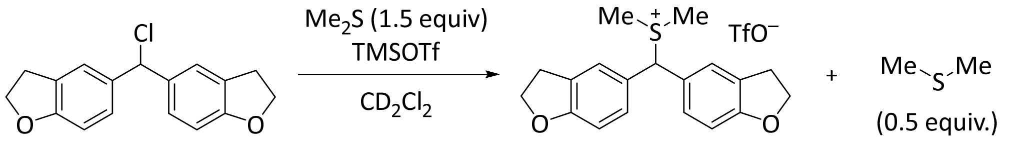

4g-Cl | (**4g-SMe$_2$**)·TfO$^-$ c = 0.107 M | **2a** c = 0.053 M

The ^{1}H NMR spectrum (400 MHz) of the thus prepared solution acquired at –80 °C showed quantitative consumption of **4g-Cl** and exclusive formation of the trialkylsulfonium triflate (**4g-SMe_2**)·TfO^-.

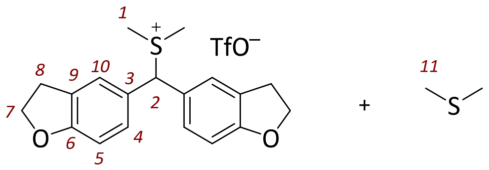

(**4g-SMe_2**)·TfO^- **2a**

^{1}H NMR (400 MHz, –80 °C, CD_2Cl_2): δ 7.39 (s, 2 H, H-10), 7.30 (s, 2 H, H-4), 6.75 (d, J = 3.7 Hz, 2 H, 5-H), 5.91 (s, 1 H, 2-H), 4.55 (s, 4 H, 7-H), 3.16 (s, 4 H, 8-H), 2.78 (s, 6 H, 1-H), 2.02 (s, 6 H, 11-H).

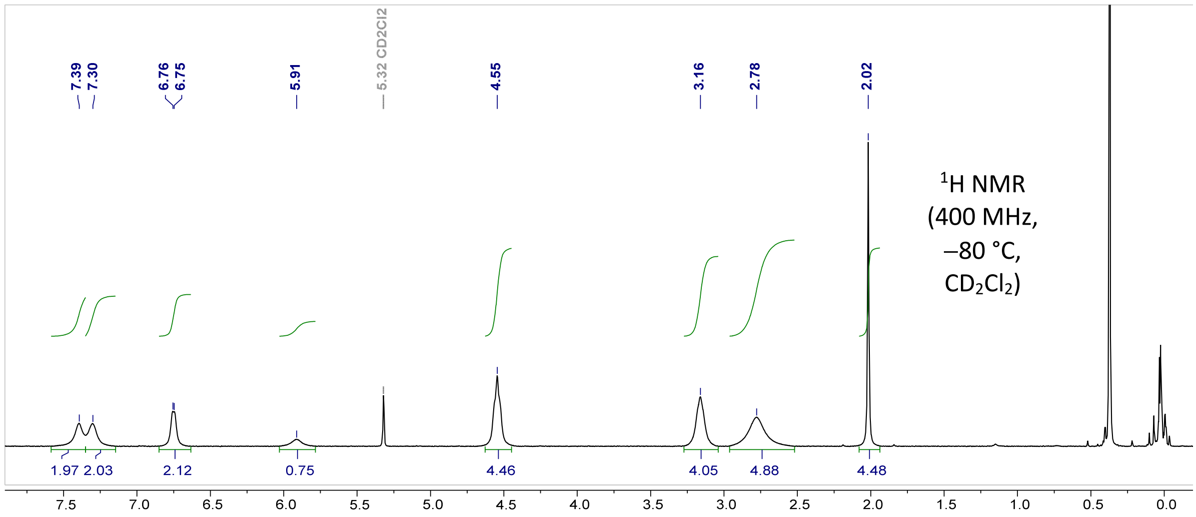

Broad resonances indicated the dynamics of exchange between free and bound Me_2S, which triggered us to further investigate the kinetics of the exchange reaction by temperature-dependent ^{1}H NMR spectroscopy. Hence, this sample was used to acquire ^{1}H NMR spectra (400 MHz) at variable temperature (Figure S1). Line shape analysis of broadened resonances was performed by manual fitting with simulated spectra generated by the DNMR6 algorithm of *iNMR* software to determine $k_{rev}(T)$.[S5]

Eyring activation parameters were determined by applying the temperature-dependent rate constants k_{rev} (s^{-1}) in the Eyring equation:

$$\ln (k_{rev}/T) = -\Delta H^{\ddagger}/R \times 1/T + \ln (k_B /h) + \Delta S^{\ddagger}/R$$

Determined rate constants and activation parameters are gathered in Table S1.

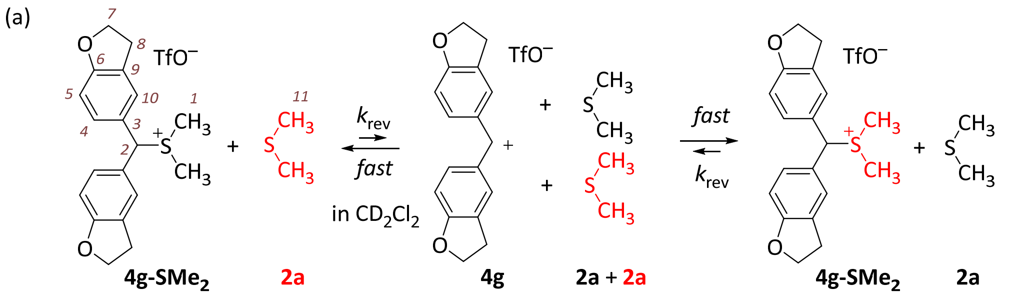

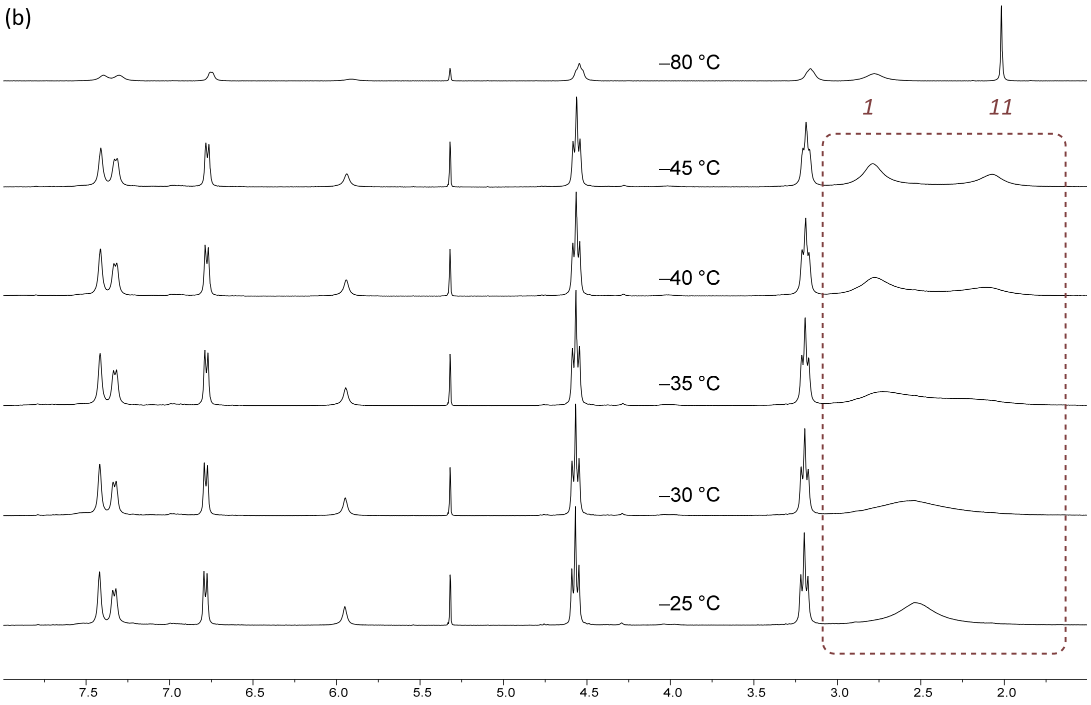

Figure S1. (a) Dimethylsulfide exchange between **4g-SMe$_2$** and free **2a**. (b) Temperature-dependent ^{1}H NMR spectra of the reaction mixture (in CD_2Cl_2). The dashed frame indicates the part of the spectrum evaluated in the line shape analysis (DNMR6 algorithm) for the determination of $k_{rev}(T)$.

Table S1. Rate constants k_{rev} and Eyring activation parameters for **4g-SMe$_2$** (in CD_2Cl_2).

T (°C)	k_{rev} (s^{-1})
–45	110
–40	200
–35	380
–30	780
–25	1.40×10^3

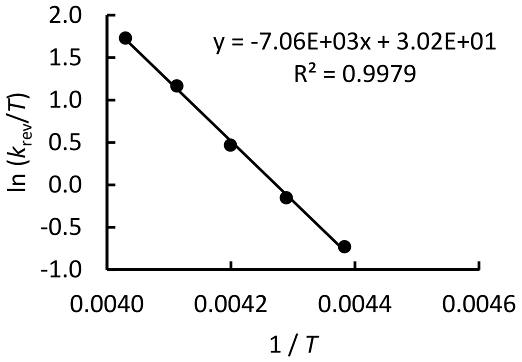

Activation parameters for k_{rev} (at 20 °C):
$\Delta G^{\ddagger}$ = 43.1 kJ mol^{-1}
$\Delta H^{\ddagger}$ = 58.7 kJ mol^{-1}
$\Delta S^{\ddagger}$ = 53.3 J K^{-1} mol^{-1}
k_{rev}(20 °C) = 1.3×10^5 s^{-1}

3.2 Dynamics of the Me_2S exchange in a CD_2Cl_2 mixture of bis(4,4′-methoxy)benzhydryl dimethylsulfonium triflate (4e-SMe$_2$ OTf$^-$) and dimethyl sulfide (2a)

(PJ142)

Generation of (**4e-SMe$_2$**) triflate in CD_2Cl_2 solution: **4e**-Cl (0.107 M), dimethylsulfide (**2a**, 0.118 M, 1.1 equiv.), and trimethylsilyl triflate (TMSOTf, 0.111 M, 1.05 equiv.) were dissolved in 0.7 mL CD_2Cl_2.

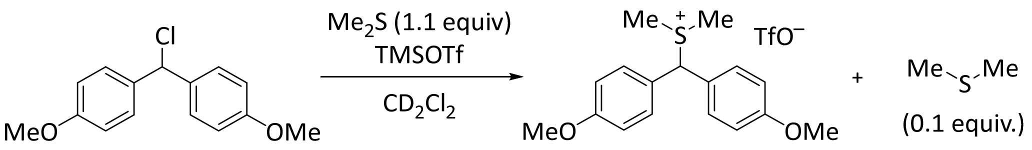

4e-Cl | **(4e-SMe$_2$)**·TfO$^-$ c = 0.107 M | **2a** c = 0.011 M

The ^{1}H NMR spectrum (400 MHz) of the thus prepared solution acquired at –60 °C showed quantitative consumption of **4e**-Cl and exclusive formation of the trialkylsulfonium triflate (**4e-SMe$_2$**)·TfO$^-$.

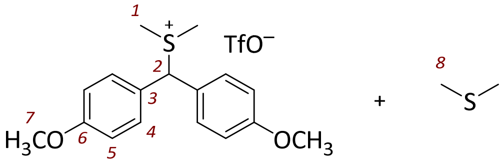

(4e-SMe$_2$)·TfO$^-$ **2a**

^{1}H NMR (400 MHz, CD_2Cl_2, at –60 °C): δ 7.49 (d, J = 8.7 Hz, 2 H, 4-H), 6.93 (d, J = 8.7 Hz, 2 H, 5-H), 5.86 (s, 1 H, 2-H), 3.75 (s, 6 H, 7-H), 2.74 (s, 6 H, 1-H), 2.05 (s, 6 H, 8-H).

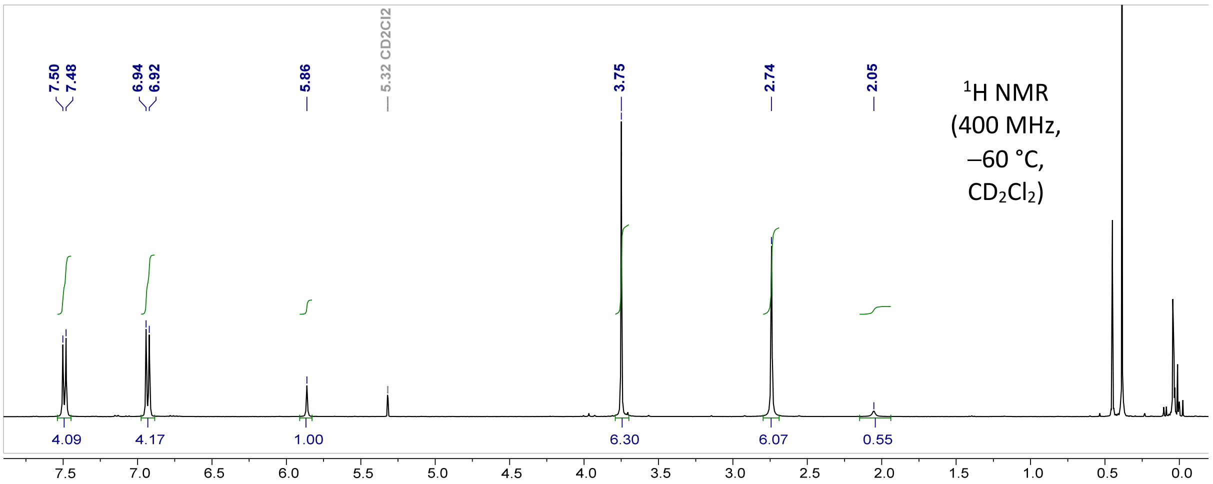

Broad resonances for 1-H and 8-H indicated the dynamics of exchange between free and bound Me_2S (**2a**), which triggered us to further investigate the kinetics of the exchange reaction by temperature-dependent ^{1}H NMR spectroscopy. Hence, this sample was used to acquire ^{1}H NMR spectra (400 MHz) at variable temperature (Figure S2). Line shape analysis of broadened resonances was performed by manual fitting with simulated spectra generated by the DNMR6 algorithm of *iNMR* software to determine $k_{rev}(T)$.[S5]

Eyring activation parameters were determined by applying the temperature-dependent rate constants k_{rev} (s^{-1}) in the Eyring equation:

$$\ln (k_{rev}/T) = -\Delta H^{‡}/R \times 1/T + \ln (k_B/h) + \Delta S^{‡}/R$$

Determined rate constants and activation parameters are gathered in Table S2.

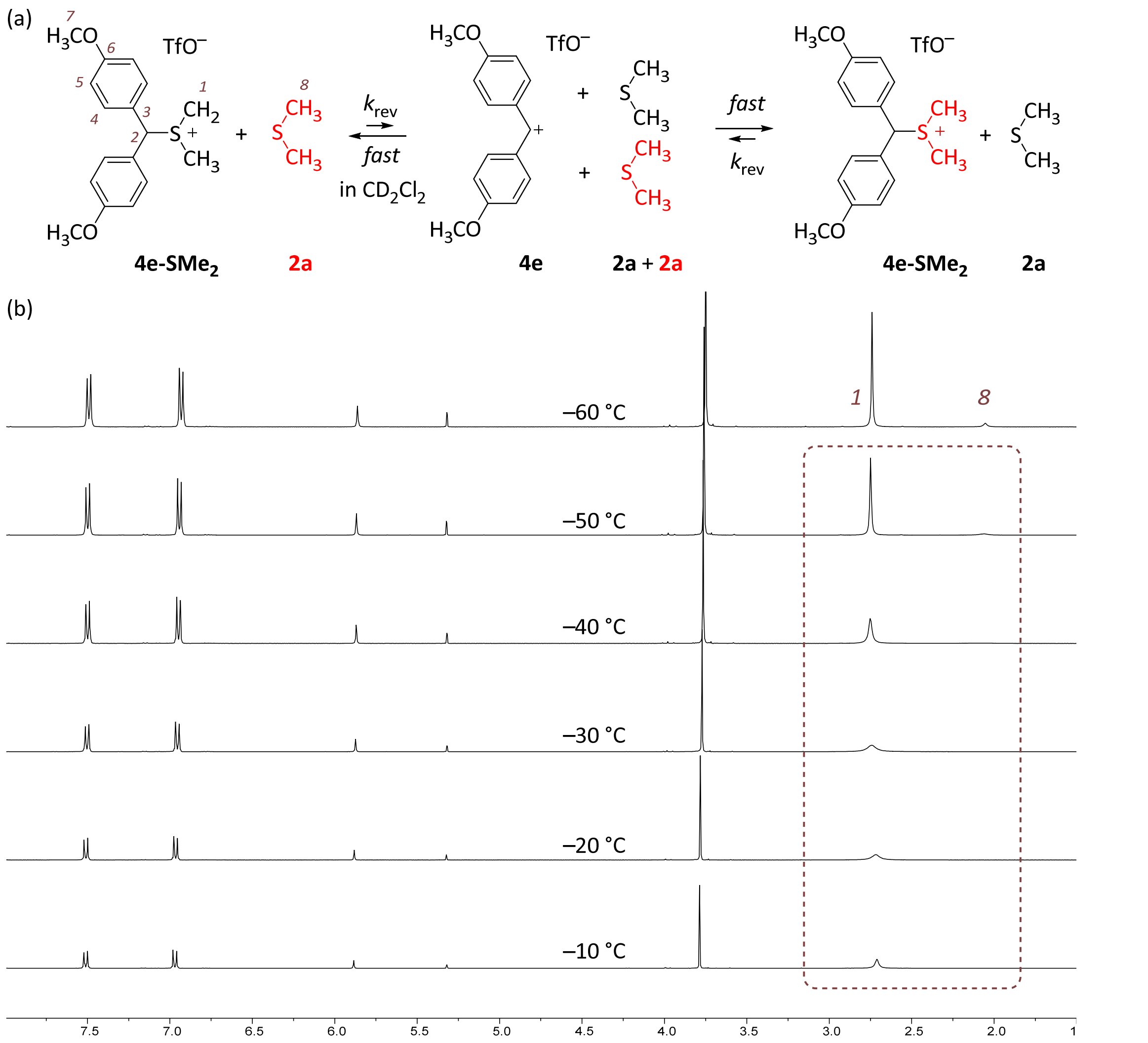

Figure S2. (a) Dimethylsulfide exchange between **4e-SMe₂** and free **2a**. (b) Temperature-dependent ^{1}H NMR spectra of the reaction mixture (in CD_2Cl_2). The dashed frame indicates the part of the spectrum evaluated in the line shape analysis (DNMR6 algorithm) for the determination of $k_{rev}(T)$.

Table S2. Rate constants k_{rev} and Eyring activation parameters for **4e-SMe₂** (in CD_2Cl_2).

T (°C)	k_{rev} (s^{-1})
−50	3.50
−40	19.0
−30	90.0
−20	245
−10	880
0	3.70×10^3

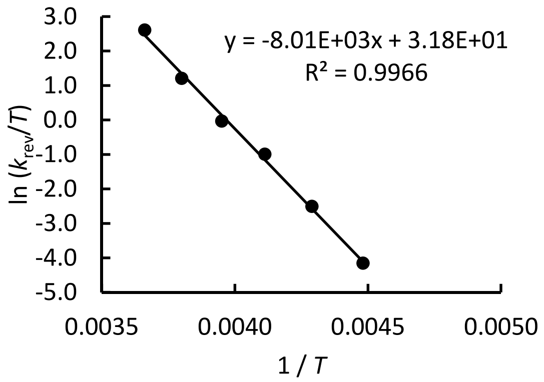

Activation parameters for k_{rev} (at 20 °C):
$\Delta G^{\ddagger}$ = 47.0 kJ mol^{-1}
$\Delta H^{\ddagger}$ = 66.6 kJ mol^{-1}
$\Delta S^{\ddagger}$ = 66.6 J K^{-1} mol^{-1}
k_{rev}(20 °C) = 2.6×10^4 s^{-1}

6. Copies of ^{1}H and ^{13}C NMR spectra of Lewis adducts

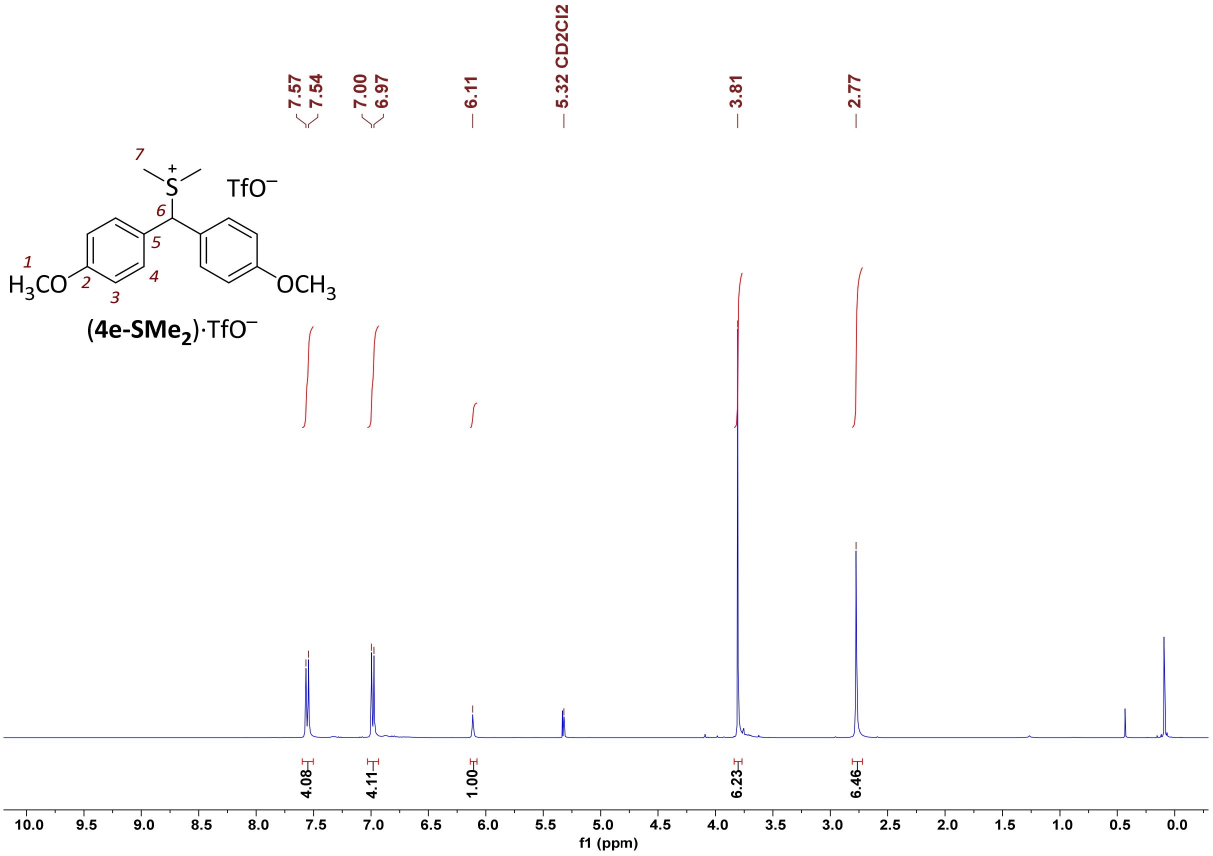

(PJ304)

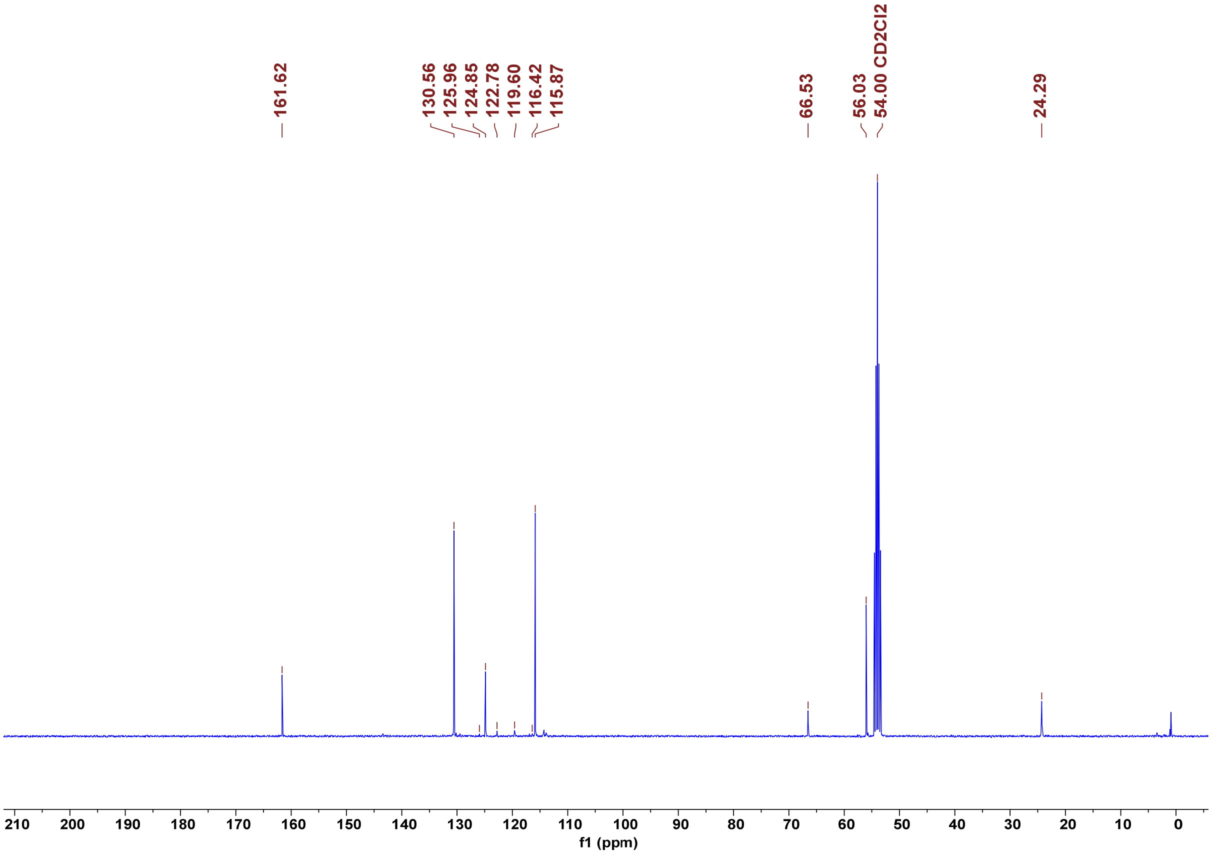

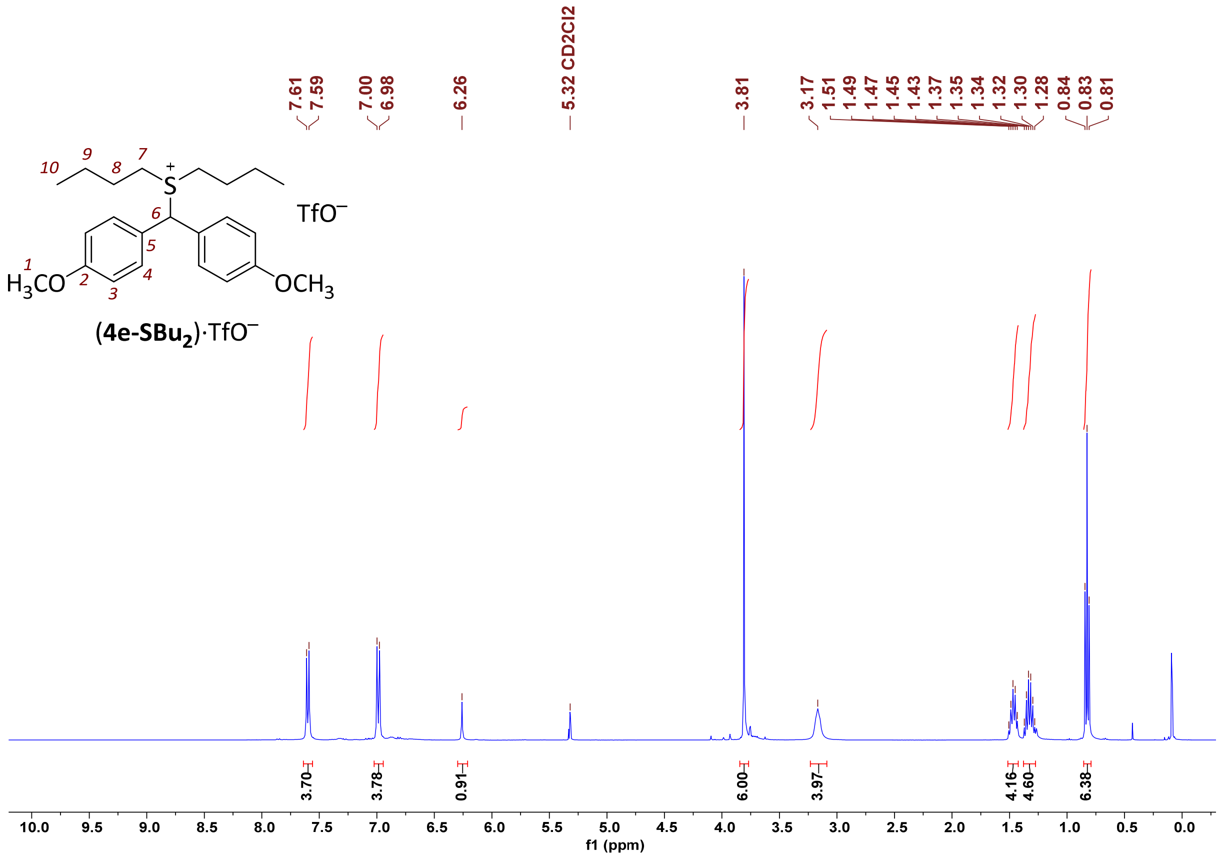
10
9
8
7
S
6
5
4
3
2
1
H3CO
OCH3
TfO−
(4e-SBu2)·TfO−
7.61
7.59
7.00
6.98
6.26
5.32 CD2Cl2
3.81
3.17
1.51
1.49
1.47
1.45
1.43
1.37
1.35
1.34
1.32
1.30
1.28
0.84
0.83
0.81
3.70
3.78
0.91
6.00
3.97
4.16
4.60
6.38
10.0 9.5 9.0 8.5 8.0 7.5 7.0 6.5 6.0 5.5 5.0 4.5 4.0 3.5 3.0 2.5 2.0 1.5 1.0 0.5 0.0
f1 (ppm)

(PJ205)

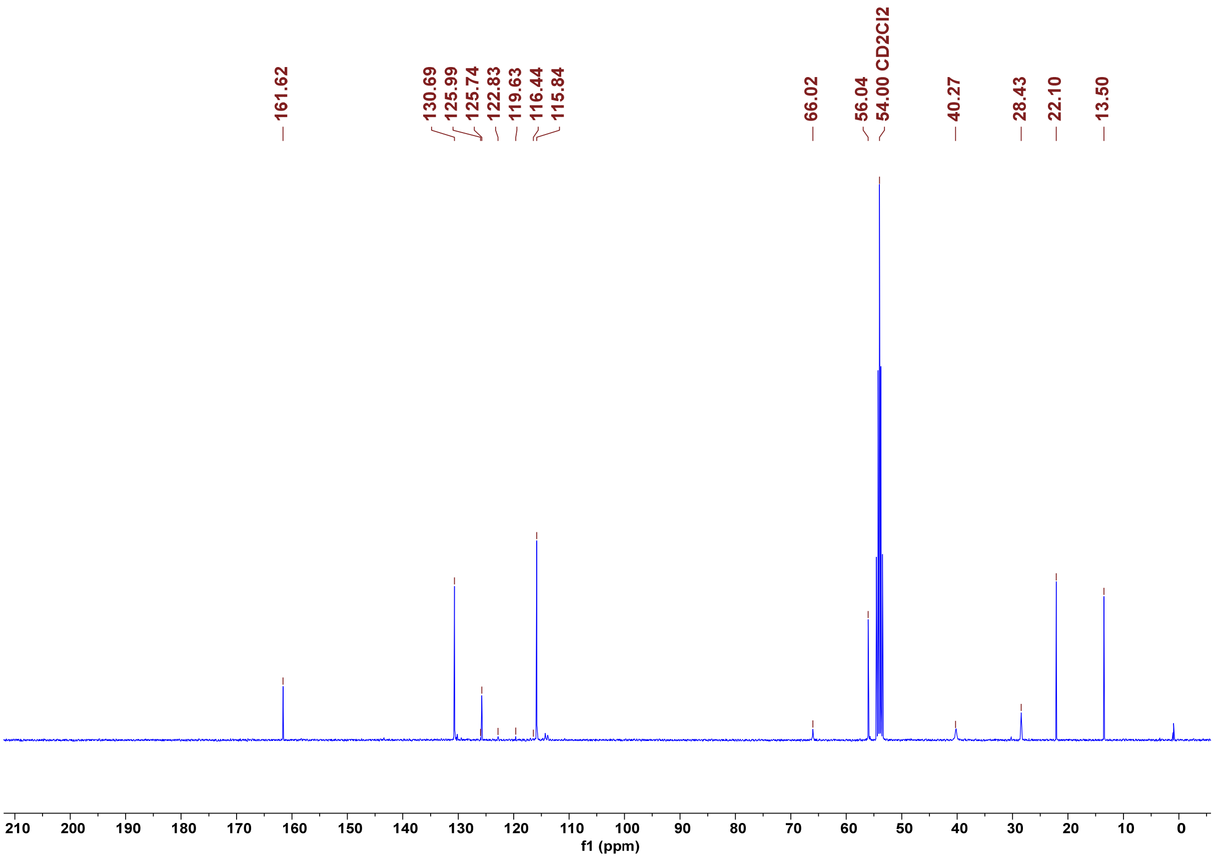
161.62
130.69
125.99
125.74
122.83
119.63
116.44
115.84
66.02
56.04
54.00 CD2Cl2
40.27
28.43
22.10
13.50
210 200 190 180 170 160 150 140 130 120 110 100 90 80 70 60 50 40 30 20 10 0
f1 (ppm)

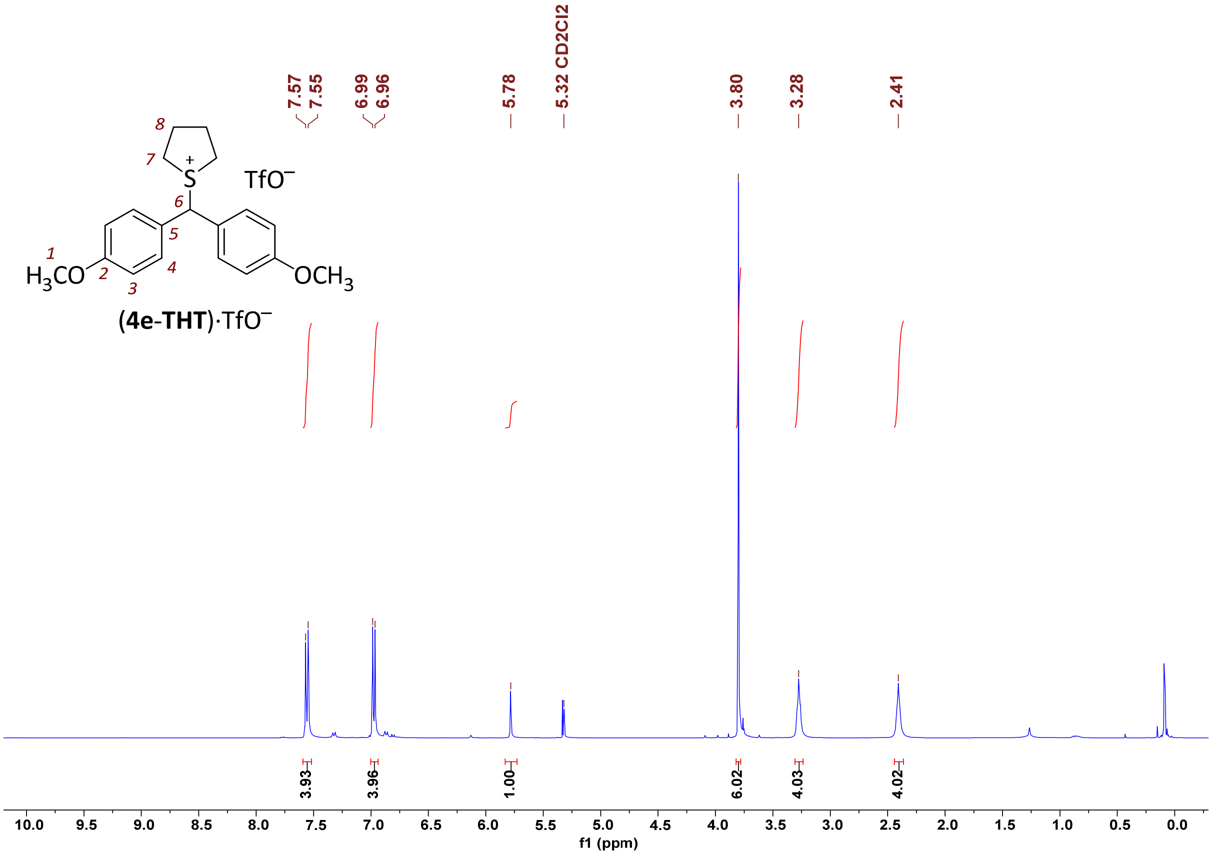
7.57
7.55
6.99
6.96
5.78
5.32 CD2Cl2
3.80
3.28
2.41
8
7
6
5
4
3
2
1
TfO⁻
H3CO
OCH3
(4e-THT)·TfO⁻
3.93
3.96
1.00
6.02
4.03
4.02
10.0 9.5 9.0 8.5 8.0 7.5 7.0 6.5 6.0 5.5 5.0 4.5 4.0 3.5 3.0 2.5 2.0 1.5 1.0 0.5 0.0
f1 (ppm)

(PJ306)

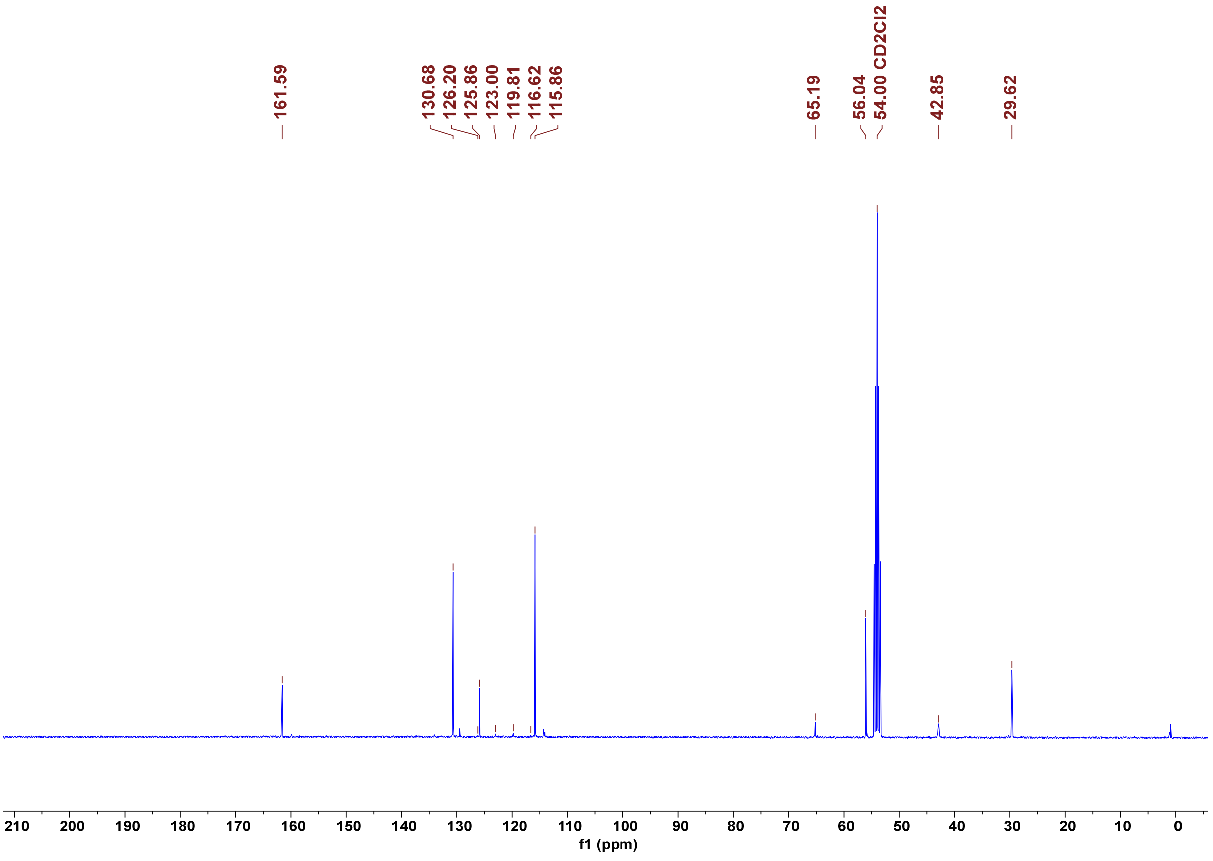
161.59
130.68
126.20
125.86
123.00
119.81
116.62
115.86
65.19
56.04
54.00 CD2Cl2
42.85
29.62
210 200 190 180 170 160 150 140 130 120 110 100 90 80 70 60 50 40 30 20 10 0
f1 (ppm)

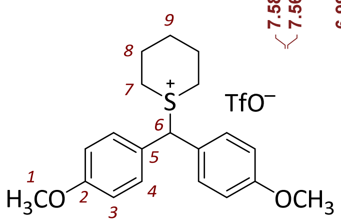

(4e-THTP)·TfO−

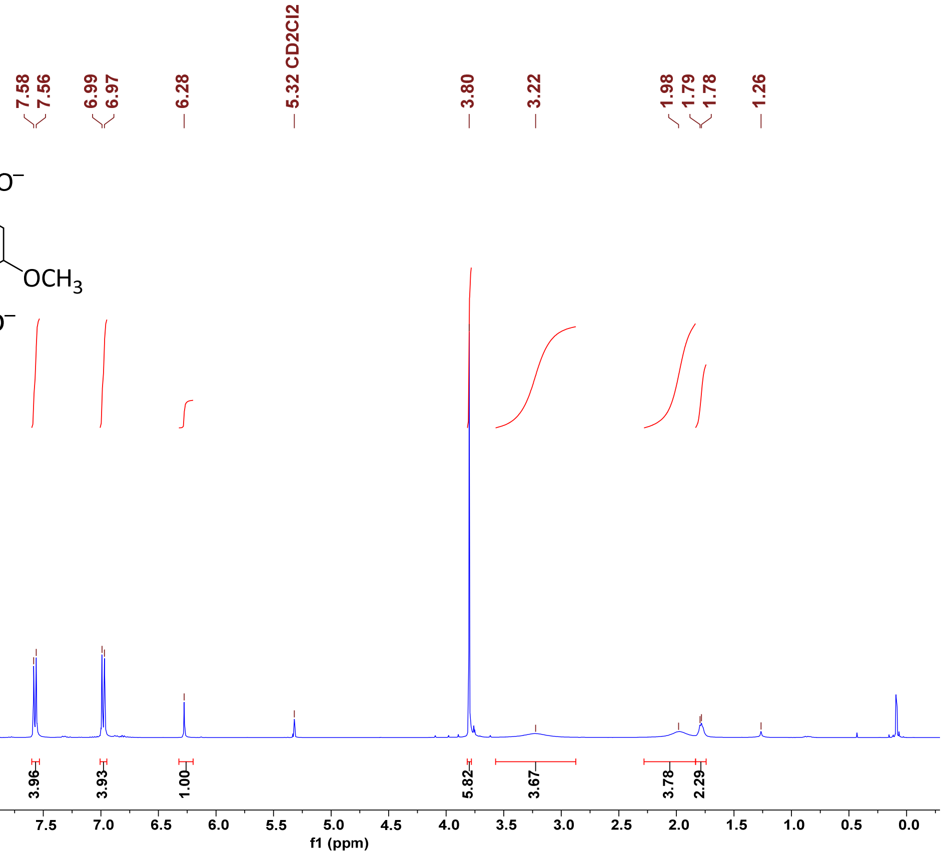

(PJ307)

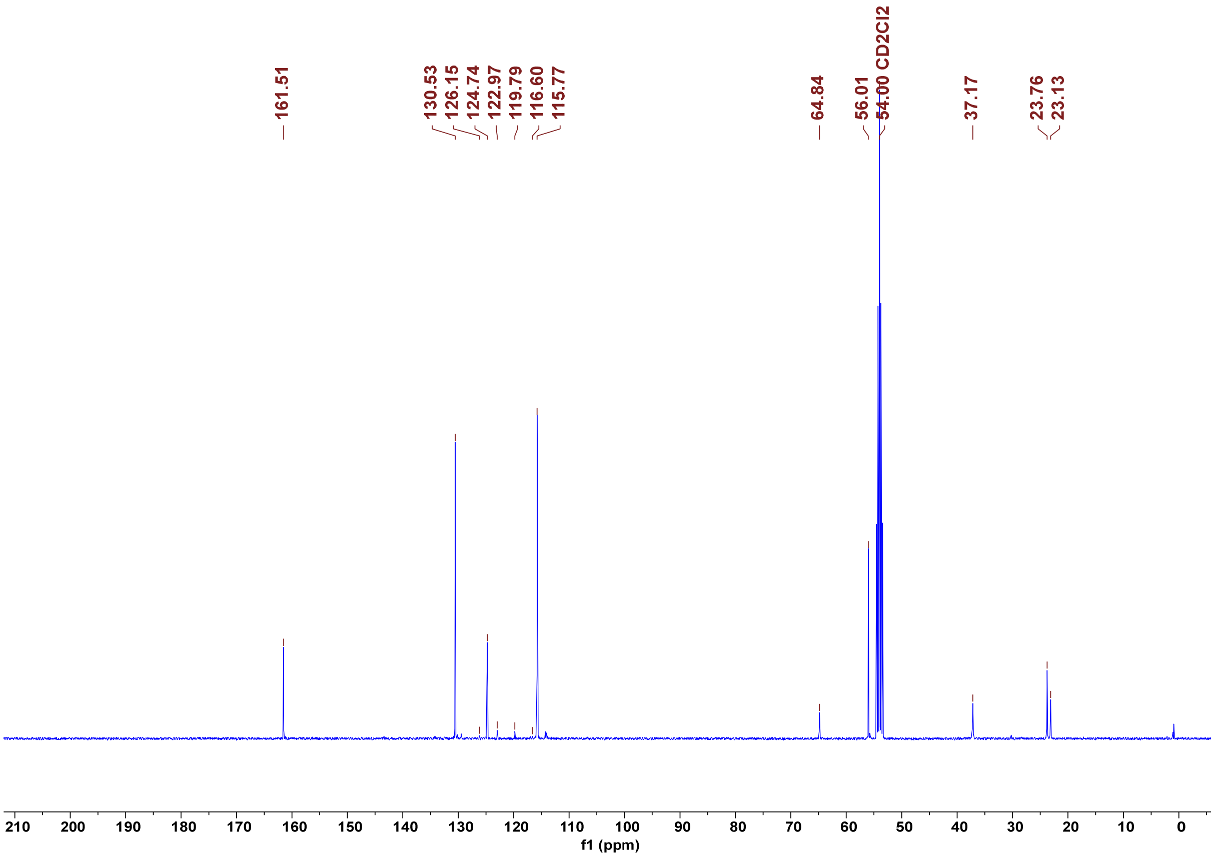

7. References

[S1] J. Ammer, C. Nolte, K. Karaghiosoff, S. Thallmair, P. Mayer, R. de Vivie-Riedle, H. Mayr, *Chem. Eur. J.* **2013**, *19*, 14612-14630.

[S2] (a) B. Denegri, A. Streiter, S. Juric, A. R. Ofial, O. Kronja, H. Mayr, *Chem. Eur. J.* **2006**, *12*, 1648-1656; *Chem. Eur. J.* **2006**, *12*, 5415. (b) A. King, A. Doepner, D. Turton, D. M. Ciobota, C. Da Pieve, A.-C. Wong Te Fong, G. Kramer-Marek, Y.-L. Chung, G. Smith, *Org. Biomol. Chem*. **2018**, *16*, 2986-2996.

[S3] G. R. Fulmer, A. J. M. Miller, N. H. Sherden, H. E. Gottlieb, A. Nudelman, B. M. Stoltz, J. E. Bercaw, K. I. Goldberg, *Organometallics* **2010**, *29*, 2176-2179.

[S4] (a) J. Ammer, C. F. Sailer, E. Riedle, H. Mayr, *J. Am. Chem. Soc.* **2012**, *134*, 11481-11494. (b) J. Ammer, H. Mayr, *J. Phys. Org. Chem*. **2013**, *26*, 956-969.

[S5] Mestrelab Research, *iNMR for Windows* (version 6.1.8, http://www.inmr.net), 2018.
